# Comparing Aquatic Environmental DNA, Microscopy and Sedimentary DNA to Investigate Cyanobacterial Community Dynamics Across a Trophic Gradient

**DOI:** 10.1007/s00248-026-02806-2

**Published:** 2026-06-30

**Authors:** Robin Michael Crucitti-Thoo, Tümer Orhun Aykut, Agnieszka Rudak, Iwona Jasser

**Affiliations:** Department of Ecology and Environmental Conservation, Faculty of Biology, Biological and Chemical Research Centre, University of Warsaw, ul. Żwirki i Wigury 101, Warsaw, 02- 089 Poland

**Keywords:** Cyanobacteria, Freshwaters, Metabarcoding, EDNA, Eutrophication, Invasive species, SedDNA

## Abstract

As a result of increasing temperatures and anthropogenic stressors, freshwater biomonitoring indicates that toxigenic and non-native cyanobacterial species are increasing globally. Because of the high-effort nature of traditional microscopy, aquatic Environmental DNA (eDNA) metabarcoding as a form of biomonitoring is becoming commonplace. While eDNA has been found to complement microscopic phytoplankton analysis, it has also been demonstrated that recent eDNA from surficial sediments (sedDNA) can also be a powerful tool to monitor a variety of organisms including benthic and planktonic microorganisms. However, comparisons between all three methods are rare. Therefore, we compared cyanobacterial metabarcoding data derived from aquatic eDNA and sedDNA to cell density and biomass determined by traditional planktonic microscopic analysis on samples taken from more than 20 lakes in the Masurian and Suwałki Lakelands of north-east Poland; an area composed of thousands of lakes with diverse morphologies, land-use histories, and varying trophic states. We found that there was a high degree of within method (eDNA/sedDNA, cell density/biomass) correspondence between the approaches, with trophic changes plausibly driving general patterns in taxa leading to these underlying agreements. Between methods, cell density proved to be more closely associated with eDNA, but was not found to be statistically significant. We postulate that cell numbers are a more similar metric to sequence read numbers than biomass, but that unique qualities of each measure mean that the approaches examined are complementary, rather than redundant. We detected several invasive taxa, which appear to have increased in abundance along the trophic gradients, and postulate that the accumulating nature of sediments may prove useful in detecting invasive taxa, particularly in the low-abundance early introductory stages.

**Supplementary Information:**

The online version contains supplementary material available at https://doi.org/10.1007/s00248-026-02806-2.

## Introduction

Cyanobacteria are a cosmopolitan, ecologically significant, and prevalent feature of the majority of Earth’s lakes, occurring in planktonic, benthic, and biofilm habitats and appearing across a range of compositions and abundance, with planktonic forms in particular generally varying due to differences in nutrients [1]. While nutrient status is not the only driver [2], rapid increases are causing unprecedented levels of cyanobacterial biomass [3], and alterations to the taxonomic structure and function of cyanobacterial communities worldwide [4]. A key concern raised due to the proliferation of cyanobacteria are the various negative impacts they can have on aquatic ecosystems as a result of the biosynthesis of toxins [5], oxygen depletion [6], ecosystem disruption, [7], alterations to the taste and odor of drinking water, and the economic costs and public health risks that arise [8]. Consequently, given the speed of changes observed there are strong motivations to be able to cheaply, accurately, and quickly identify cyanobacteria on an ongoing basis to monitor the rate of change and mitigate their effects [6].

In comparison to traditionally applied light microscopy, metabarcoding studies using Environmental DNA (eDNA) have been shown to be beneficial, since while the start-up costs of equipment and the sequencing itself can be high, in comparison to the number of expert working-hours needed to accurately identify and quantify microorganisms such as cyanobacteria using microscopy, DNA based studies can often be performed relatively quickly and cheaply compared to traditional microscopic analysis [9]. Moreover, metabarcoding approaches can often detect higher numbers of taxa, thus providing a complementary approach to light microscopy [10, 11]; a method that can determine essential metrics such as algal biomass, but may miss or misidentify particularly small taxa that tend to dominate in low nutrient environments [12, 13], as well as underestimate buoyant taxa which are particularly prevalent at high trophic levels [9]. Metabarcoding is also subject to a number of limitations such as database issues [12], and quantification and amplification biases [14], many of which can be introduced throughout the pipeline from sampling to analysis [15]. Therefore, given the strengths and weaknesses of each approach [13], a large number of investigations have been undertaken to determine the correspondence between methods across a range of trophic, environmental, and geographical gradients. Generally, they have been found to differ significantly at lower taxonomic levels, while broader taxonomic comparisons, especially in lakes of higher tropic status, showed greater congruence. This being most likely due to higher rates of accuracy in identification at genus level and above using microscopy, as well as the lower relative contribution of picocyanobacteria in high nutrient systems [9, 16].

Given the time intensive nature of sample collection and analysis required of continuous biomonitoring – whether by eDNA or microscopy – alternative repositories of biological material may prove useful, particularly for detecting invasive taxa which are often sporadically present and in low numbers. There is increasing evidence that using surface sediment samples (sedimentary eDNA or sedDNA) – which contain DNA derived both from settled pelagic organisms [17] and microbial communities inhabiting the sediments themselves [18] – has a number of advantages over taking a large number of recurrent water samples for ecological monitoring [19]. Given the protection afforded to DNA bound to sediment as it accumulates [20], it stands to reason that sediments may provide a longer time-integrated record of the biosphere, rather than a single ‘snapshot’ from a water derived sample [21, 22]. For example, Turner et al. [23] demonstrated that carp DNA was more concentrated in sediments than in the water column, and present for up to three months longer. In this respect, sedDNA may prove useful for the monitoring of the establishment of invasive cyanobacteria, since only consistent ecological monitoring can confidently detect organisms in the early stages of biological invasion [24]. However, general understanding of the response of the sediment microbial communities to changes in trophic status is relatively limited [25]. For terrestrial and aquatic sediments it is generally considered that micro-to-macro scale habitat variations are the main determinants of community structure, rather than large scale trends such as variations in lake trophic status [25, 26]. This is in contrast to planktonic cyanobacteria in lake waters, which have been shown to vary along large-scale geographic and trophic gradients [2]. While the diversity of phytoplanktonic communities may be expected to peak at intermediate productivity or disturbance levels [27], in prokaryotic phytoplankton such as cyanobacteria, it is possible that increases in trophic status lead to increases in taxonomic alpha diversity up until hypertrophy [28, 29]. This may be attributable to increases in the overall number of taxa that can occupy a given location as nutrient levels rise, without necessarily reducing the total number of taxa, although the beta diversity among lakes may decline with increased trophic status due to community homogenization [4, 30].

To date relatively few investigations have taken place that directly compare eDNA to sedDNA [31]. Murray et al. [32] compared marine biodiversity of eDNA and sedDNA, determining that eukaryotic community composition was significantly different, with higher levels of heterogeneity in sediments sampled at the same locations. Notwithstanding spatial heterogeneity of biological communities in lake sediments [33, 34], it is likely that sedDNA and aquatic eDNA will both respond to changes along shared trophic or ecological gradients [35]. Moreover, in locations currently experiencing biological invasions, both water and sediments are potential eDNA repositories for biological monitoring of taxa that have been observed to increase in prevalence in nutrient rich lakes [36].

Therefore, increasing the knowledge base on how lacustrine surface sediment dwelling cyanobacteria vary between a range of lakes, and whether that variation corresponds to more frequently applied aquatic eDNA metabarcoding or light microscope based approaches, will help shed light on the effects of increased nutrient status on sedimentary communities, which is an often overlooked aspect of human/environment interactions. Given the widespread application of these methods across trophic, ecological and geographic gradients [2, 37, 38], our overall rationale was that by conducting a methodological comparison within an explicit ecological framework, we aimed to compare their correspondence under realistic ecological conditions. Moreover, considering the potential for using sedDNA as a monitoring tool for invasive cyanobacteria, which are often sporadically present in very low numbers during establishment [39], and generally appear in lower relative abundance in nutrient poor systems [36], we aimed to evaluate the sensitivity of these environmental DNA approaches for detecting invasive taxa by comparing a range of lakes spanning a trophic gradient. With these comparisons allowing us to assess which approach (if any) improves the detection of invasive taxa.

The Masurian and Suwałki Lakelands of north-east Poland contain thousands of lakes with varying trophic states. While many experience intense eutrophication, others have relatively high water quality [40]. Within the past decade, concerns have been raised about the detection of cyanobacteria considered to be invasive or range-expanding in Central and Eastern Europe due to historically low occurrences, recent rapid spread, and their ability to establish and proliferate [41, 42]. These concerns have been compounded by these organisms’ preferences for elevated temperatures and nutrients, which have been observed to be increasing in the region [43, 44]. Additionally, high levels of tourism and the interconnected nature of the Great Masurian Lake System increase the likelihood of the spread of invasive taxa. Considering our overall aim to assess the correspondence between methods in natural conditions, we hypothesise that the mix of taxa in cyanobacterial assemblages will largely depend on lake trophic status, with each method generally corresponding within each lake, and that this correspondence will hold as cyanobacterial assemblages track trophic status across the gradient. On this basis, we hypothesised that:

1. Microscopy, aquatic eDNA and sedDNA metabarcoding are expected to demonstrate congruent cyanobacterial community structures across lakes despite methodological differences.
2. Metabarcoding-derived datasets are expected to correspond more closely with one another than with microscopy-derived datasets, and vice versa. Microscope derived cell counts are expected to correspond more closely to metabarcoding reads than biomass.
3. Within analyses constrained by environmental variables, all methods will respond to increasing trophic status;
4. Alpha diversity is expected to increase with Carlson’s Trophic Status Index (TSI), with positive correlations among methods, although diversity estimates are expected to differ systematically among methodological approaches. SedDNA is additionally expected to show elevated alpha diversity due to the inclusion of benthic taxa.
5. Beta diversity patterns are expected to correspond across methods, reflecting common environmental structuring of cyanobacterial communities;
6. In lakes where invasive species have been detected, sedDNA will show higher reads and/or relative abundances of invasive taxa than eDNA due to accumulated genetic material.

## Methods

### Study Sites

**Fig. 1:**
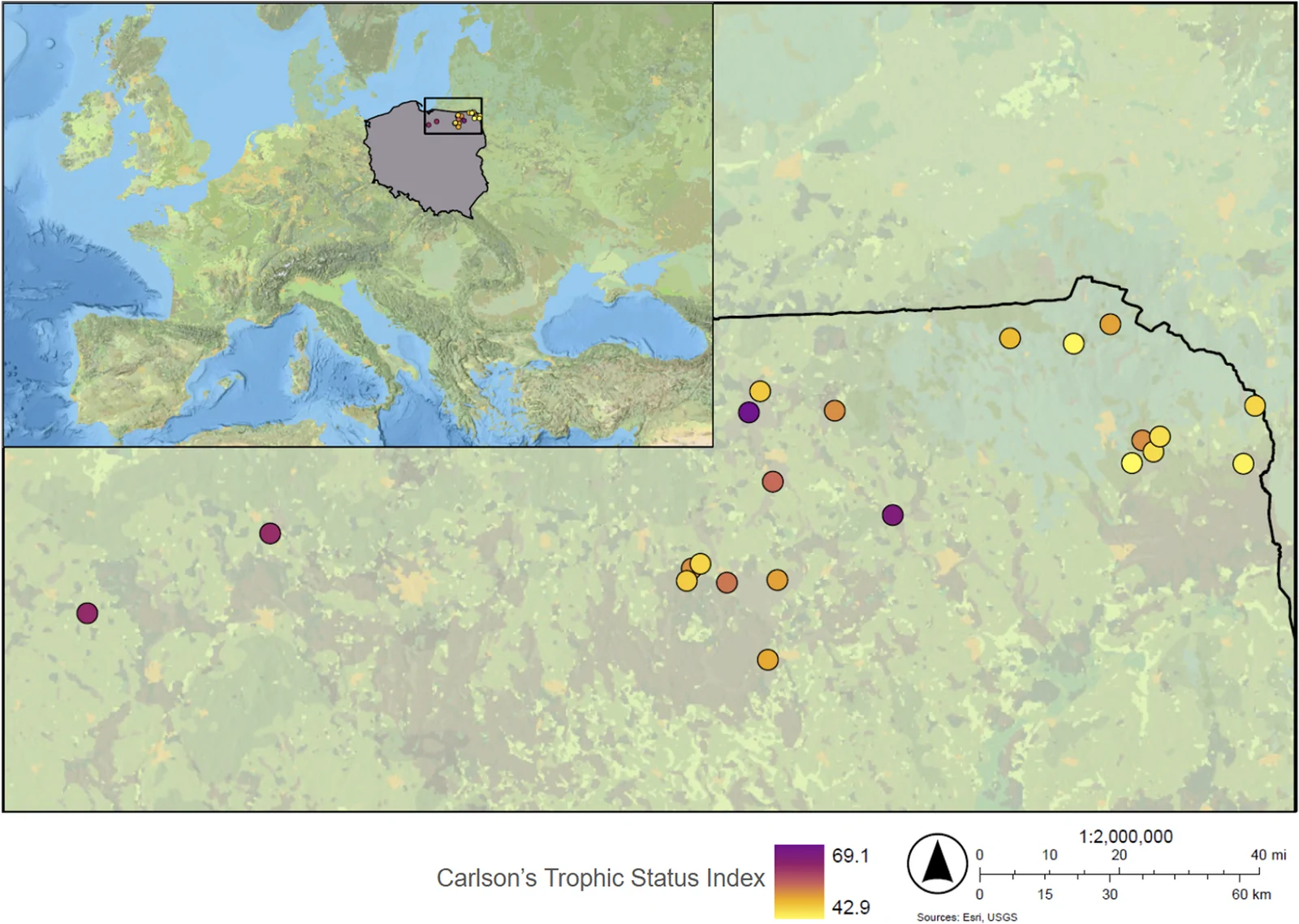
A map showing the sampling locations within Poland. Lakes are coloured by Carlson’s Trophic State Index (TSI) [46]. Mapped using ArcGIS Online [50]

Twenty two lakes were sampled (Fig. 1, Supplementary Spreadsheet), which were identified on the basis of previous investigations as lying along a trophic gradient [45]. Water quality of each lake was separately confirmed using Carlson’s Trophic State Index (TSI) [46]. To provide a framework to assess the congruence between methods in the present study, we chose the mean of Secchi disc TSI, chlorophyll *a* TSI, and Total Phosphorus TSI, as an integrated measure of trophic state on a common numerical scale. This was chosen as a standardized and reproducible measure of trophic state, integrating water transparency, phytoplankton biomass, and nutrient availability [47].

### Sampling

Samples for all analyses were collected within a four day sampling window during August, 2023. Water samples for DNA analysis and microscopy were collected at the same time, then sediment samples were taken, excluding three sediment samples which were obtained in the previous sampling season (August, 2022). Water samples were taken in the centre of each lake, or in the case of the largest lakes in the pelagic zone. Sediment samples were collected using a UWITEC gravity corer at the same location as water samples, the upper part of the core was extruded from the core liner and a subsample of around 2 cm^2^ was removed from the top ~ 2 cm of the cores into sterile falcon tubes, following a modified sampling protocol of Turner et al. [23]. When not possible to take sediment cores, falcon tubes were used to collect sediments by hand from suitable undisturbed locations at the lake shores. All sediment samples were stored at − 20℃ until DNA extraction. Based on our previous investigations at two sites from this study, Lake Żabińskie and Lake Głębokie, as well as sediment accumulation rates commonly reported for temperate lakes [48] the upper ~ 2 cm of the cores is likely to represent ~ 5–10 years of accumulation.

In the field, temperature, pH, dissolved oxygen, and electrical conductivity were measured using a YSI 556 probe. Transparency was measured with a Secchi disc. Integrated epilimnion water was sub-sampled for phytoplankton and DNA analysis. Samples for phytoplankton analysis were fixed and stained with Lugol’s solution and formaldehyde (36%) to a final concentration of 1% and stored at 4℃ [49]. Samples for DNA analysis were transferred to sterile plastic containers. At the field station 200–250 ml was filtered onto 0.2 μm polycarbonate (PC) membrane filters and stored at − 20℃ (SI Sect. 1 – Sampling Locations). Separate containers were collected for the determination of the following environmental parameters: Non-Purgeable Organic Carbon (NPOC; mg/L) and Total Nitrogen (TN; mg/L) measured by combustion using Multi N/C 3100 apparatus. Total Phosphorus (TP; mg/L) which was determined following nitric acid digestion up to 230 °C followed by measurements on a SAN++^®^ Series continuous flow analyzer. Ammonium (N-NH₄⁺; mg/L), nitrate (N-NO_3_^−^; mg/L), orthophosphate (P-PO₄^3−^; mg/L), and sulfate (S-SO_4_^2−^; mg/L) which were determined spectrophotometrically using the same analyzer.

### Microscopy

Light microscopy followed the Utermöhl method [51] with identification performed using several taxonomic keys [52–55]. Biomass calculations are fully described in [46, 56]. All microscopic analyses were conducted by T.A.

### DNA Extraction and Sequencing

DNA was extracted from DNA filters or approximately 250 mg of sediment using the EURx Soil DNA Purification Kit following the manufacturer’s instructions. DNA filters were additionally thawed from − 20 ℃ and flash frozen at −80 ℃ to destroy cell walls prior to extraction. Next-Generation Sequencing was performed using a NovaSeq 6000 platform on DNA extracts using tagged CYA primers with pair-end mode of 2 × 250 cycles. This primer pair amplifies an approximately 400 bp long fragment of the V3 and V4 hypervariable regions of 16S rRNA [17] (SI Sect. 1).

### Bioinformatic Processing

Sequencing reads were processed in QIIME2 [57]. Primers were trimmed with Cutadapt [58], and sequences were denoised with DADA2 to determine ASVs [59]. Taxonomy was assigned using a custom naive-bayes taxonomic classifier trained on the SILVA database [version: 138 [60]] using the CYA primer pair. Post-classification filtering removed non-cyanobacterial sequences, organelles and features with less than 100 occurrences. More detailed parameter settings are provided in SI Sect. 1.

ASVs were collapsed to genus level, since microscopic analysis usually does not resolve to species level [9]. Therefore, comparing microscopic results to genus level taxonomic assignments is more appropriate, also taking into account limited resolution when identifying closely related species using 16S rRNA [61]. To verify the taxonomy, a representative sequence with the highest frequency from each collapsed grouping was used in a BLAST query [62]. Genus level identities were accepted at ≥ 97% identity. We also used the curated online Cydrasil3 database [63] to verify the taxonomic position of all taxa following Khomutovsta et al. [64]. In cases where BLAST results were ambiguous, < 97% identity, or if there were mismatches between SILVA, BLAST, and Cydrasil placements, we examined the probability scores, identity values, and likelihood weight ratios on a taxon-by-taxon basis. Since phylogenetic placement in a curated reference tree can provide improved taxonomic resolution [65], but these databases do not contain all taxa, we decided that this would be the most robust approach. If there was still uncertainty, then these taxa were reported at the family or order level and excluded from analyses at the appropriate taxonomic levels.

To allow for comparison between datasets, ASVs and taxa identified by microscopy were put into the same taxonomy on the basis of genus level classifications in AlgaeBase [66] and identifications merged into single taxa with the abundances summed [67].

On the basis of previous studies, we determined that the following invasive taxa had the possibility of being detected: *Raphidiopsis raciborski* and *Raphidiopsis mediterranea* [41], *Sphaerospermopsis aphanizomenoides* [68], *Cuspidothrix issatschenkoi* [69], *Chrysosporum bergii*, *Chrysosporum minus*, and *Anabaena minderi* [70]. These taxa have been observed since the mid-twentieth century in parts of Europe, for example *Raphidiopsis raciborski* was first noted in Greece in 1937, followed by an increasing number of records across Central and Northern Europe in recent decades [42]. The pattern of spread is supported by either an absence of records, or very low contributions to phytoplankton communities up until the past few decades, and appears to be increasing in geographical extent [56]. We used a targeted approach for the detection of these species. Taxa were collapsed to species-level followed by a BLAST query as described above. Given the limited resolution when identifying closely related species using metabarcoding, we only considered a species detected if it had 100% sequence identity for the target taxon. In instances where 100% identity was shared with non-target taxa, we used a combined approach of SILVA and Cyrdasil phylogenetic placement for validation. To avoid removal of rare taxa after 100 the occurrences threshold, we additionally checked all annotated reads prior to filtering for target taxa.

#### Statistical Analysis

All statistical analyses were performed in R [71]. Four lakes were excluded from the analysis due to issues with the sampling probes and poor sequencing results. All detected invasive taxa were included in the analyses.

To assess the overall congruence between the multivariate patterns among the lakes, we performed Principal Component Analysis (PCA) separately on the eDNA, sedDNA, cell density (cells/L), and biomass (mg/L) datasets at the genus, family, and order taxonomic levels. Following MacKeigan et al. [9] we extracted PCA scores into a matrix containing the first 3 PCs from each PCA. We then used the RV coefficient *coeffRV()* from the FactoMineR package [v 2.12, [72]] to make pairwise comparisons between these configurations at each taxonomic level. The RV coefficient quantifies similarity between multivariate configurations by measuring the closeness of two sets of points in their ordination spaces [73] and can be used to assess how similarly two separate methods, such as eDNA reads and cell numbers identified to genus level, describe the patterns of differences across the lakes. As an additional check on the relationship between the main gradients in community structure and trophic status, we performed Spearman’s rank correlations between TSI and the first three principal component (PC) axes from each PCA.

Redundancy Analyses (RDA) were performed to explore the relationships between the cell density, biomass, eDNA, and sedDNA at genus level, and environmental parameters. A correlation check was performed to remove collinear variables, and variance inflation factors (VIF Scores) were calculated to ensure no variables had a VIF > 5 with the “vif.cca” *vegan* function. Several environmental parameters were excluded based on collinearity tests, or because they were consistently close to or below detection limits. Additionally, after running multiple models substituting TN, Secchi depth, or TSI, TSI was retained as an integrative measure of trophic status. Permutation-based ANOVAs (9,999 permutations) were used to assess the overall significance of the multi-variable RDA models and the significance of each variable with adjusted R^2^ values. Additionally, we used the “ordiR2step” *vegan* function to perform forward selection permutation analysis with R2scope = FALSE [74] and 9,999 permutations.

To assess the similarity of the ordination configurations derived from multivariable RDAs, we made pairwise comparisons using Procrustes analysis [75] with the *procrustes()* and *protest() vegan* functions. Procrustes analysis can be used to quantify the similarity between different datasets by aligning them in multivariate space by translating, scaling, and rotating one ordination to match another. It then calculates the distance between corresponding points, and has been used to compare community datasets derived from different methods including between data derived from metabarcoding and microscopy [76, 77]. We then performed a Procrustes permutation test using the *protest() vegan* function to assess the similarity between RDA site coordinates. This was done under the assumption that a significant fit between ordinations would indicate that each pair of ordinations captured the same main ecological gradients and the responses of each dataset to the same environmental variables [78].

In both PCA and RDA, aquatic eDNA and sedDNA ASV read counts, as well as cell counts and biomass values from microscopy data were Hellinger transformed using the *decostand()* function [79, 80]. Other data were z-score scaled.

Alpha diversity, taxonomic richness, Shannon’s diversity index and Pielou’s evenness were calculated using the *vegan* package on untransformed abundance data. To compare diversity metrics generated by each approach, we used the Friedman test. We used Paired Wilcoxon signed-rank tests to compare Shannon’s diversity index and Pielou’s evenness scores; p-values were adjusted using the Benjamini–Hochberg (BH) correction. To gauge the direction of the differences, we calculated median paired differences as the median of lake-by-lake differences between methods, e.g. median(*Shannon*_*sedDNA, i*_ *- Shannon*_*eDNA, i*_), where *i* represents each lake, and also summed the total number of lakes that had higher Shannon’s diversity estimates. Additionally, Spearman’s correlation was used to check for correlations between pairs of diversity metrics between methods, and between diversity metrics and TSI, which was calculated on the basis of phytoplankton assemblages and environmental parameters [46].

Between lake beta diversity was assessed by calculating Bray-Curtis dissimilarity using *vegdist()* on relative abundances normalised with *decostand (“total”)*. Pairwise Mantel tests were performed on the derived Bray–Curtis distances to correlate the distance matrices with 9,999 permutations with Benjamini–Hochberg adjusted p-values. Unconstrained ordinations of Bray–Curtis distances were made using the *capscale(~ 1*,* distance = “bray”)* function, corresponding to Principal Coordinates Analysis (PCoA), to visualise patterns in community composition. Following Muñoz-Yáñez et al. [81] we used the *vegan envfit()* function to identify the taxa most strongly associated with the compositional gradients determined by Bray-Curtis PCoA.

For invasive taxa, to determine the relative sensitivity of each DNA repository, sediment: water ratios were calculated for each detected taxon and lake as the relative read abundance (%) in sediment divided by that in water (% sediment/% water).

## Results

### Cyanobacterial Community Structure

**Fig. 2:**
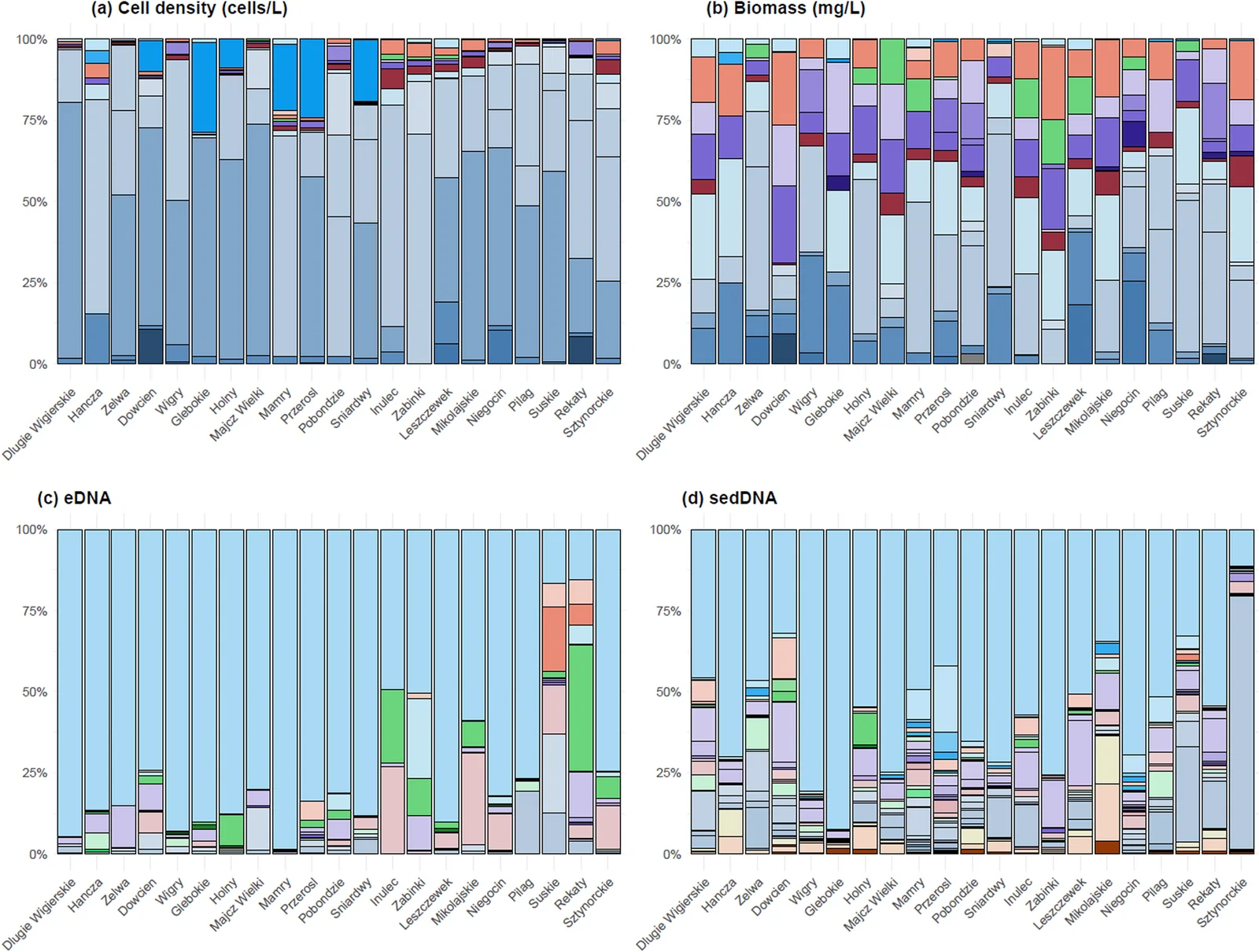

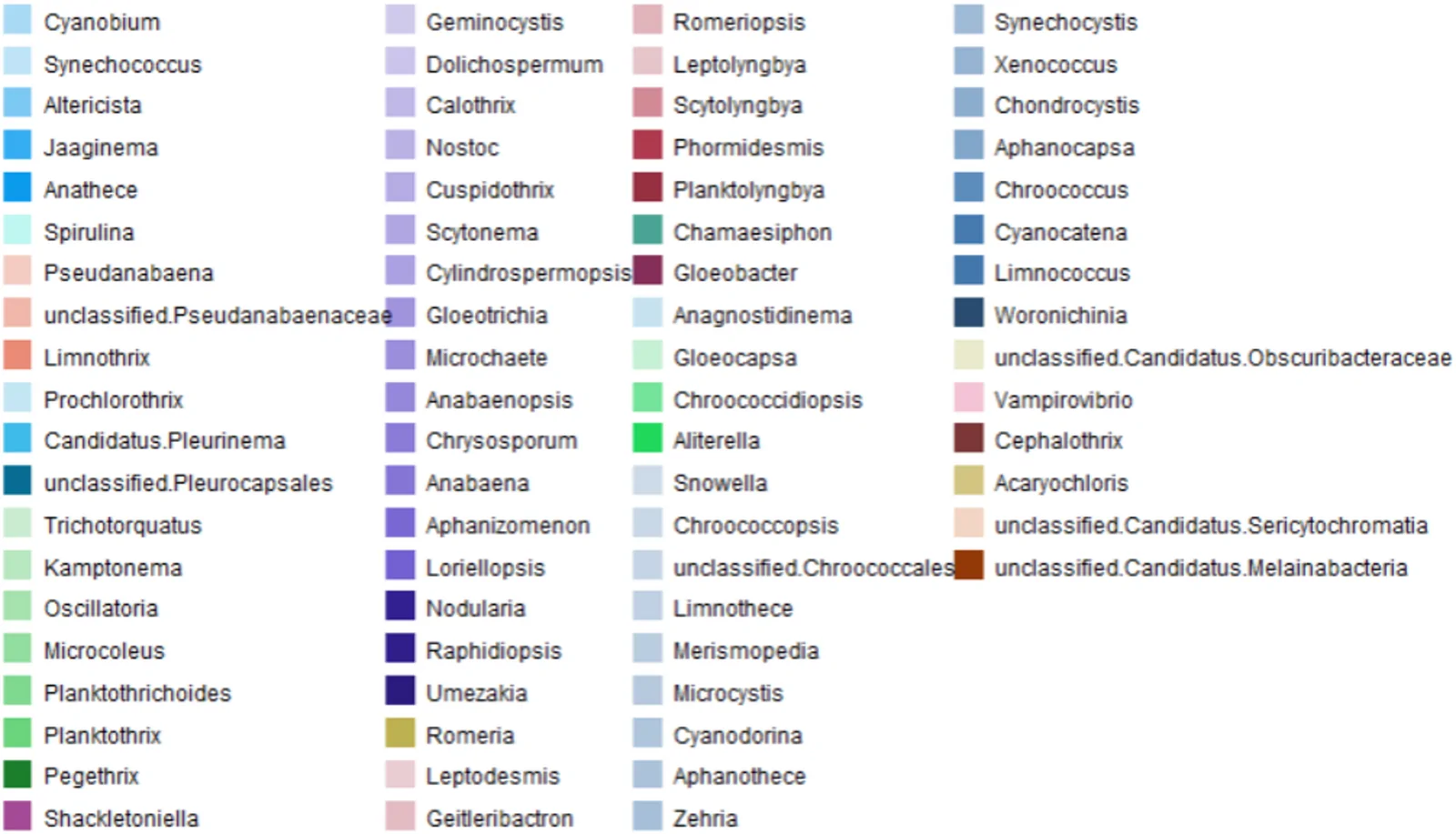
Genus-level relative abundance of cyanobacteria after taxonomic harmonisation using AlgaeBase. Lakes are ordered from left to right according to their TSI. (**a**) Microscopy cell density (cells/L), (**b**) microscopy biomass (mg/L), (**c**) aquatic eDNA relative abundance (% of total reads), and (**d**) sedimentary DNA relative abundance (% of total reads)

A total of 69 cyanobacteria taxa were identified using microscopy, cell density ranged from 2,586.8 to 590,647.9 cells/L per lake, biomass ranged from 0.242 to 23.072 mg/L. When grouped to genus level, 30 genera were identified (Fig. 2). After bioinformatic processing, 102 genus level cyanobacterial ASVs were obtained (77 eDNA; 95 sedDNA). Twenty-five were unique to sedDNA and 6 unique to eDNA. Further grouping on the basis of AlgaeBase resulted in 62 genera in sedDNA and 53 in eDNA, 13 and 4 of which were unique to sedDNA and eDNA, respectively. Lake Brzozolasek sedDNA only had 68 reads, compared to eDNA reads of 285,408. Since we were unable to compare datasets, this site was removed for further analysis. The top five most abundant genera in terms of microscope derived cell density were *Aphanocapsa* (48.13%), *Microcystis* (24.20%), *Merismopedia* (10.31%), *Snowella* (6.04%), and *Anathece* (3.93%). For biomass the top five were *Microcystis* (31.39%), *Anagnostidinema* (20.37%), *Aphanizomenon* (10.47%), *Limnothrix* (8.55%), and *Dolichospermum* (5.24%). The top 5 aquatic eDNA ASVs in terms of percentage were: *Cyanobium* (71.28%), *Leptolyngbya* (6.32%), *Planktothrix* (6.30%), *Dolichospermum* (3.68%), *Snowella* (3.80%); for sedDNA *Cyanobium* (57.57%), *Microcystis* (7.45%), *Cyanodorina* (4.27%), *Geminocystis* (3.88%), and *Dolichospermum* (3.33%).

### Multivariate Correspondence Between Methods

**Fig. 3:**
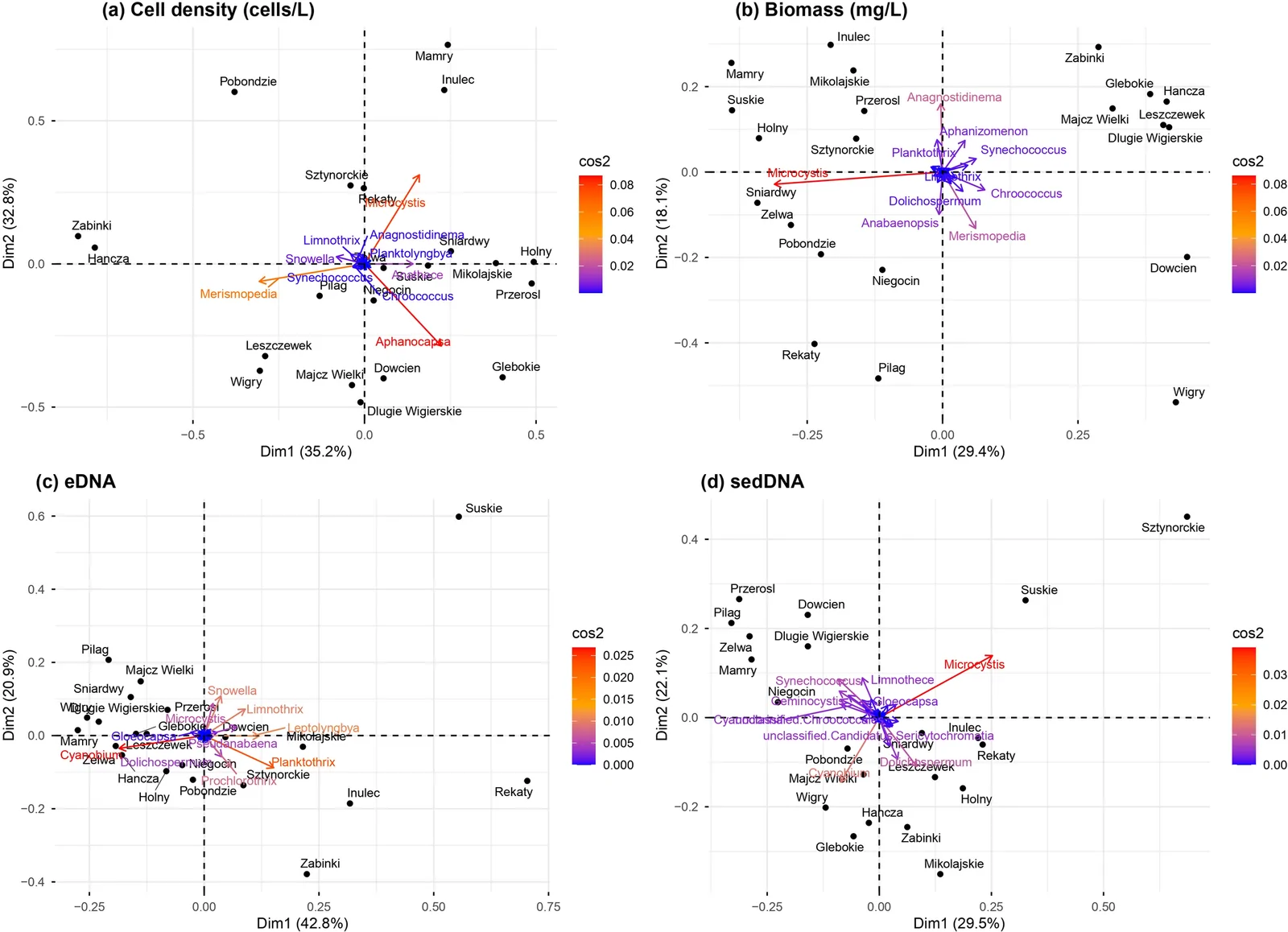
Genus level PCA biplots (**a**) Cell density (cells/L), (**b**) Biomass (mg/L), (**c**) eDNA, (**d**) sedDNA: Sites are in black with the top ten contributors to PC1 and PC2 labelled. The purple to red gradient shows the strength of contribution of a given variable to the cos2 values, representing contributions to the first two principal components

RV coefficients at the genus level (based on PCAs from Fig. 3) showed that the correspondence between the multivariate structure of the PCs within methods i.e. eDNA/sedDNA (RV = 0.29; *p* = 0.027), and cell density/biomass (RV = 0.49; p = < 0.001) were the only statistically significant agreements (SI Sect. 2). At the family level RV coefficients showed no significant agreements, although cells/biomass (RV = 0.23; *p* = 0.055) and eDNA/sedDNA (RV = 0.25; *p* = 0.056) approached significance. At the order level, no significant correspondences were found. Spearman’s rank correlations between TSI and the first principal component (PC) from each PCA found significant relationships at genus level between eDNA (𝝆 = 0.618, *p* = 0.003) and sedDNA (𝝆 = 0.454, *p* = 0.039), and borderline non-significant relationships between TSI and biomass (both 𝝆 = −0.393, *p* = 0.078). At the family and order levels only eDNA was significantly related to TSI (both 𝝆 = 0.609, *p* = 0.003). Correlations between TSI and PC2 were borderline non-significant for genus level cell density (𝝆 = 0.385, *p* = 0.085) and significant for family level sedDNA (𝝆 = 0.432, *p* = 0.050). For PC3 at the genus level cell density was significantly related to TSI (𝝆 = −0.578, *p* = 0.006).

The full RDA model performed on aquatic eDNA explained 48.25% of total variance (adj. R² = 0.094, F = 1.73, *p* = 0.044), with the greatest contribution coming from SO₄²⁻ (F = 2.35, *p* = 0.057). Forward selection using ordiR2step identified TSI and SO₄²⁻ as the main predictors with 25.68% of the variance explained (adj. R² = 0.258, F = 4.48, p = < 0.001) (see SI Sect. 3 for RDA models).

The full sedDNA RDA explained 41.08% of the variance, but was not statistically significant (F = 1.307, *p* = 0.133; adj. R² = 0.097). Forward selection using ordiR2step identified TSI as the only significant predictor, explaining 13.4% of the variance (adj. R² = 0.089, F = 2.9427, *p* = 0.005). The full cell density RDA model explained 44.44% of the variance, but was not significant (F = 1.48, *p* = 0.086, adj. R² = 0.145). OrdiR2step identified the sole significant predictor as Maximum Depth (F = 2.68, *p* = 0.030, adj. R² = 0.077) explaining 12.34% of the variance. For biomass, the full RDA model explained 41.43% of the variance, but was not significant (F = 1.314, *p* = 0.096, adj. R² = 0.099). Forward selection using OrdiR2step identified Secchi Depth (F = 2.033, *p* = 0.037, adj. R² = 0.049) as the only significant predictor, explaining 4.91% of the variance.

**Fig. 4:**
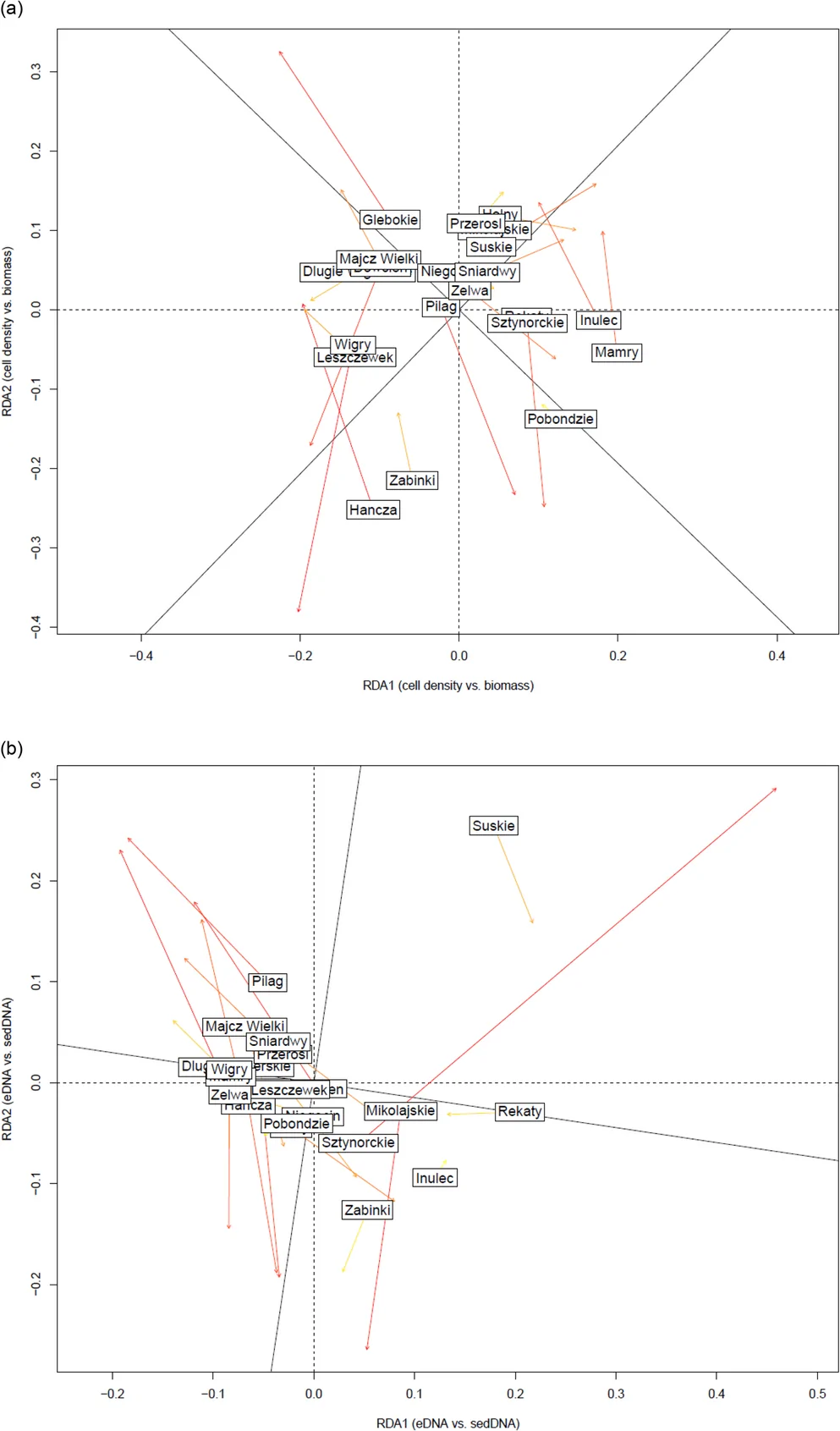

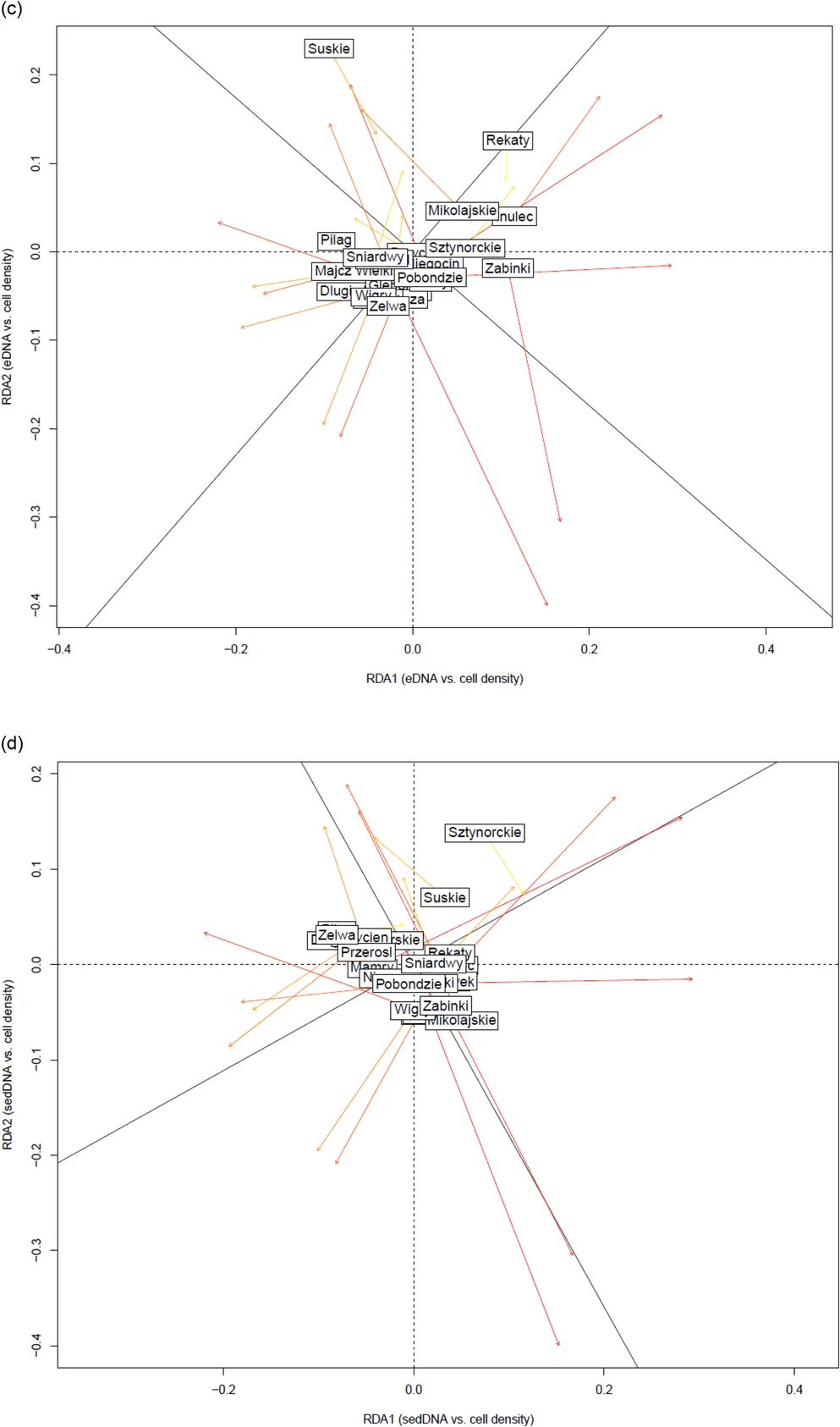

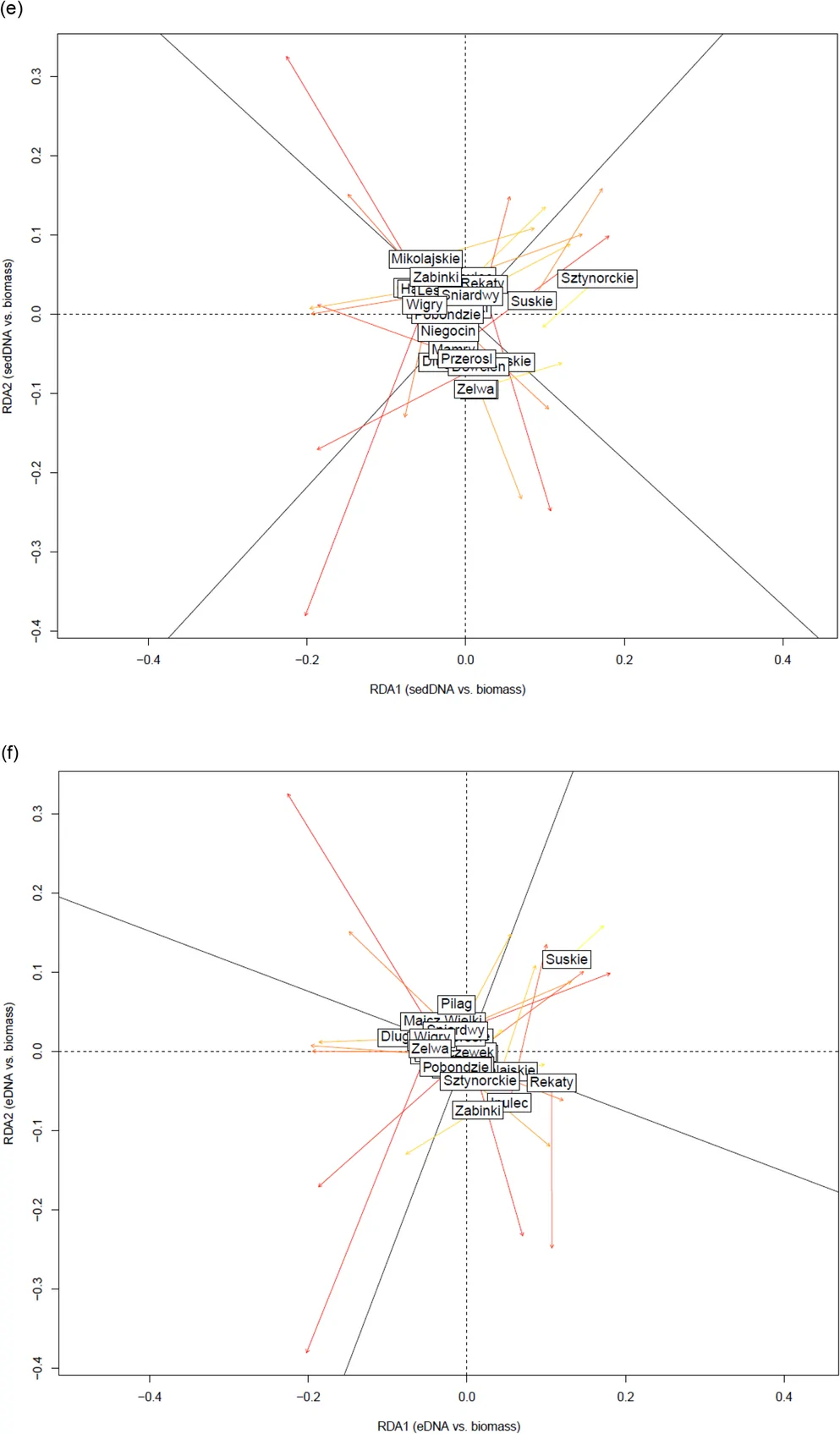
Procrustes analysis of (**a**) cell density and biomass, (**b**) eDNA and sedDNA, (**c**) eDNA and cell density (**d**) sedDNA and cell density, (**e**) sedDNA and biomass, and (**f**) eDNA and biomass. Site arrows are coloured from yellow (least different) to red (most different), connecting corresponding samples between ordinations based on residuals (given site name overlap, see also SI Sect. 4: Procrustes Residuals for lake-by-lake comparisons). Solid and dashed lines represent the orientations of the target and rotated configurations following Procrustes alignment

Procrustes analysis between full RDA models found significant relationships between eDNA/sedDNA and cell density/biomass with borderline non-significance between eDNA/cell density (Table 1; Fig. 4).

**Table 1:** Procrustes analysis between genus level RDA ordinations by sample type. The sum of squares ranges from 0–1, with 0 indicating a perfect alignment between ordinations. P-values are derived from the “protest” permutation test

Test 1	Test 2	Procrustes sum of squares	Procrustes correlation (*r*)	*p*-value
Cells	Biomass	0.589	0.641	< 0.001
eDNA	sedDNA	0.739	0.511	0.014
eDNA	Cells	0.834	0.408	0.060
sedDNA	Cells	0.896	0.289	0.370
sedDNA	Biomass	0.907	0.317	0.252
eDNA	Biomass	0.921	0.281	0.338

### Biodiversity Metrics

All methods systematically differed for Shannon’s diversity (Friedman χ²(3) = 38.886, p = < 0.001, *n* = 21 lakes) and Pielou’s evenness (Friedman χ²(3) = 45.114, p = < 0.001, *n* = 21 lakes). For Shannon’s diversity all paired Wilcoxian comparisons were significantly different at the p.adj = < 0.05 level. All paired Wilcoxian comparisons were different for Pielou’s evenness at the p.adj = < 0.005 level, excluding sedDNA/cell density (W = 86 p.adj = 0.313). SedDNA had 17/21, 16/21, and 7/21 lakes with higher Shannon’s diversity, and a paired median difference of 0.785, 0.181, and − 0.344 natural units compared to eDNA, cell density and biomass, respectively. For Pielou’s evenness, sedDNA had higher values than eDNA in 16/21 lakes, 8/21 compared to cell density, and 0/21 compared to biomass. Paired median differences were 0.209, −0.060 and − 0.334, respectively.

**Fig. 5:**
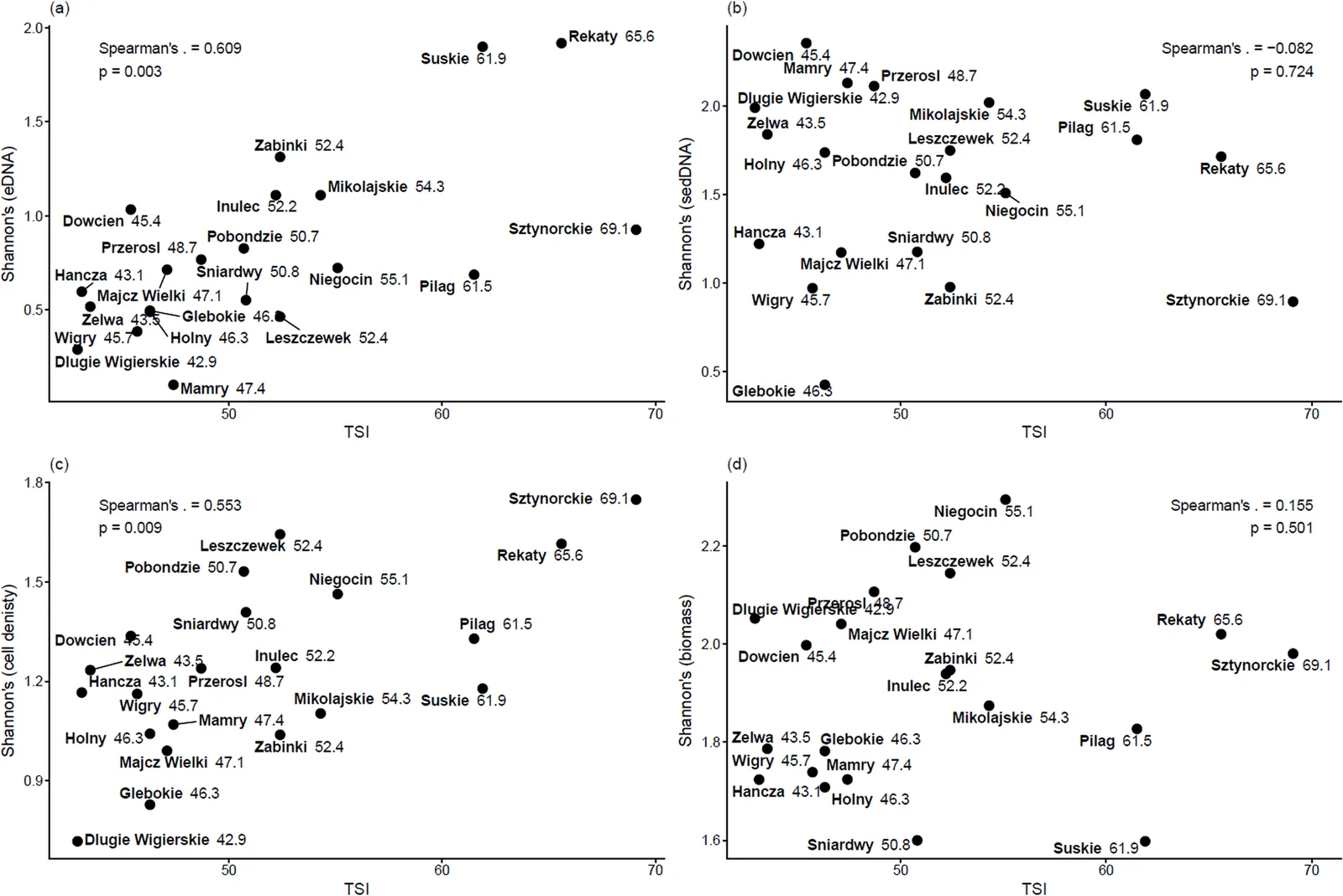

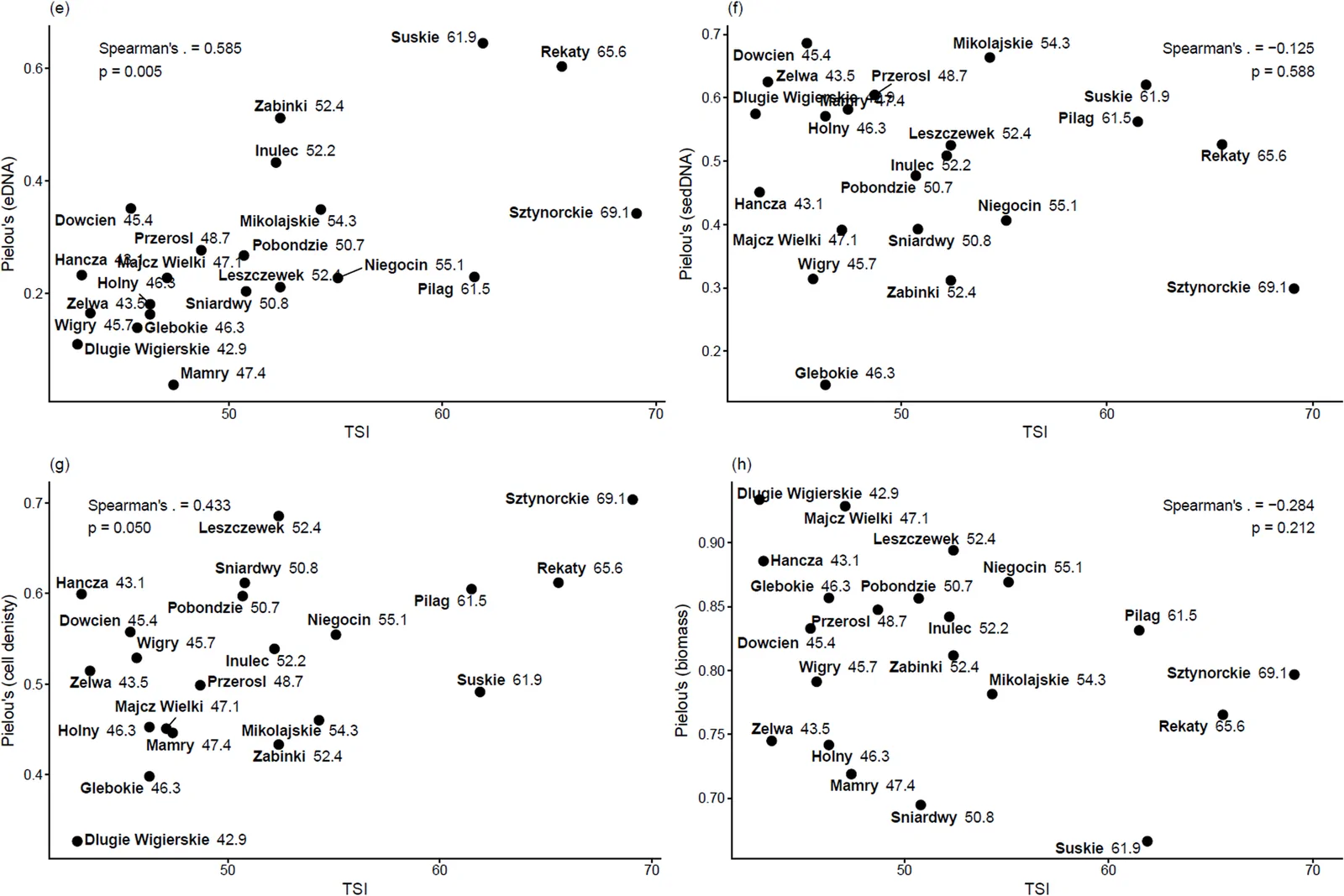
Relationships between trophic state (TSI) and alpha diversity metrics. The y-axis is scaled individually per chart. Each lake’s TSI value is shown. Spearman’s rank correlations were used to assess relationships: Shannon’s diversity: (**a**) eDNA, (**b**) sedDNA, (**c**) cell density, (**d**) biomass. Pielou’s evenness: (**e**) eDNA, (**f**) sedDNA, (**g**) cell density, (**h**) biomass

Pairwise Spearman’s correlations of Shannon’s diversity and Pielou’s evenness indices for the four methods (eDNA, sedDNA, cell density, and microscopy) were weak and inconsistent across methods with no statistically significant relationships observed, suggesting limited agreement in diversity estimates (Tables SI 5.1, 2). However, Shannon’s diversity correlations between eDNA/TSI (𝝆 = 0.609, *p* = 0.003, *n* = 21, adj.*p* = 0.034) and cell density/TSI (𝝆 = 0.553, *p* = 0.009, *n* = 21, adj. *p* = 0.047) were moderately positively correlated and statistically significant. For Pielou’s evenness eDNA/TSI were marginally non-significantally related on the basis of adjusted p-values (𝝆 =0.585, *p* = 0.005, *n* = 21, adj. *p* = 0.053). On the basis of raw p-values, cell density/TSI were also significantly related (𝝆 = 0.433, *p* = 0.049, *n* = 21, adj.*p* = 0.248) (Fig. 5; SI Sect. 5).

**Fig. 6:**
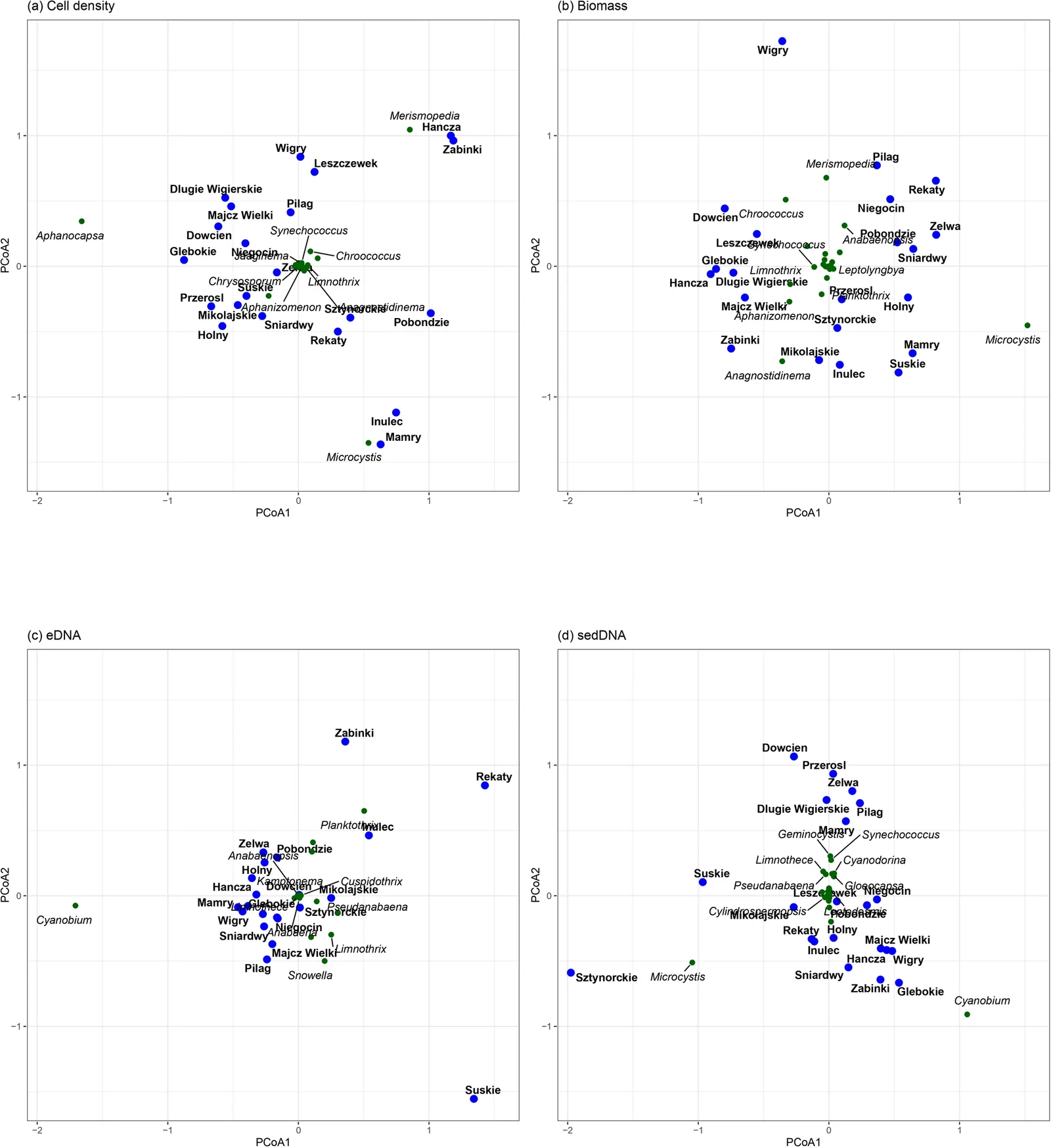
Unconstrained ordinations of Bray–Curtis dissimilarities for (**a**) cell density (cells/L), (**b**) biomass (mg/L), (**c**) eDNA, and (**d**) sedDNA. The top ten taxa based upon *envfit()* R^2^ values are labelled. Other taxa are represented by green points only. Lakes are shown as blue points

For beta diversity, calculated Bray-Curtis dissimilarity (Fig. 6.) compared using Mantel tests showed no significant relationships between any sample type or method at the distance matrix level (Mantel *r* = −0.167 to 0.057, all *p* > 0.275). On the basis of *envfit()*, multiple taxa were significantly associated with the PCoA ordinations (see SI Sect. 6 for full vector scores). Of these, for eDNA only *Cyanobium* was negatively associated with the main ordination axis with the strongest fit and highest significance. Whereas, 8 taxa typically associated with higher trophic conditions were significantly positively related. Likewise, for sedDNA *Cyanobium* had the strongest fit and significance value, followed by *Microcystis.* The strongest relationships with the main ordination axis for cell density were *Microcystis*, *Merismopedia*, with *Aphanocapsa* associated with the opposite end of the main axis. The top driver of biomass was *Microcystis*. Notably, *Aphanocapsa* was not significantly associated with the main ordination axes, while *Synechococcus* had a strong fit.

### Detection of Invasive Species

We detected invasive taxa in 14 of 21 lakes with *Sphaerospermopsis aphanizomenoides* in 3 lakes (query cover 100%, E-value 0, per. ident. 100%; clustered with *Sphaerospermopsis aphanizomenoides* in the Cyrdasil3 reference tree), *Raphidiopsis raciborskii* in 4 lakes (query cover 100%, E-Value 0, per. ident 100%; clustered with *Cylindrospermopsis raciborskii*), and *Cuspidothrix issatschenkoi* in 12 lakes (query cover 100%, E-Value 0, per. ident. 100%; clustered with *Sphaerospermopsis aphanizomenoides*) (See SI Sect. 7 for further information).

Overall, invasive taxa were detected in 13 eDNA samples and 11 sedDNA samples, 9 of 11 sediment–water sample pairs had higher relative percentages of invasive taxa from sediments (Table 2). Due to the low number of lakes in which these taxa were detected, as well as the low number of reads/relative abundances, formal statistical comparisons were not considered robust. Instead, we present sediment: water ratios of relative read abundance (%) as a descriptive measure of their relative representation between sample types.

**Table 2:** Read numbers and percentage contribution to all reads from water or sediment samples for each detected invasive ASV. Sediment-water sample pair ratio comparisons are emboldened and denoted by an asterisk (*) in the sediment sample row. Lakes are ordered from low (top) to high (bottom) TSI values

TSI	Lake	*Cuspidothrix issatschenkoi*	*Cylindrospermopsis raciborskii*	*Sphaerospermopsis aphanizomenoides*
46.30	Głebokie (eDNA)	*Not detected*	*Not detected*	3/< 0.01%
46.30	Głebokie (sedDNA)	*Not detected*	*Not detected*	*Not detected*
46.30	Hołny (eDNA)	59/0.02%	*Not detected*	*Not detected*
46.30	Hołny (sedDNA)	88/1.3%/* **52.72**	*Not detected*	*Not detected*
48.70	Przerośl (eDNA)	1287/0.78%	*Not detected*	*Not detected*
48.70	Przerośl (sedDNA)	*Not detected*	*Not detected*	*Not detected*
50.70	Pobondzie (eDNA)	63/0.04%	*Not detected*	*Not detected*
50.70	Pobondzie (sedDNA)	12/0.06%/* **1.36**	*Not detected*	*Not detected*
50.80	Śniardwy (eDNA)	18/0.01%	*Not detected*	*Not detected*
50.80	Śniardwy (sedDNA)	103/0.97%/* **151.81**	*Not detected*	*Not detected*
52.20	Inulec (eDNA)	29/0.01%	*Not detected*	*Not detected*
52.20	Inulec (sedDNA)	243/0.62%/* **57.18**	*Not detected*	*Not detected*
52.40	Leszczewek (eDNA)	*Not detected*	*Not detected*	*Not detected*
52.40	Leszczewek (sedDNA)	12/0.06%	*Not detected*	*Not detected*
52.40	Żabinskie (eDNA)	*Not detected*	*Not detected*	*Not detected*
52.40	Żabinskie (sedDNA)	26/0.17%	*Not detected*	*Not detected*
54.30	Mikołajskie (eDNA)	794/0.28%	*Not detected*	*Not detected*
54.30	Mikołajskie (sedDNA)	35/0.33%/* **1.17**	*Not detected*	*Not detected*
55.10	Niegocin (eDNA)	466/0.2%	8/< 0.01%	*Not detected*
55.10	Niegocin (sedDNA)	436/0.43%/* **2.18**	*Not detected*	*Not detected*
61.50	Piłag (eDNA)	5/0%	*Not detected*	*Not detected*
61.50	Piłag (sedDNA)	*Not detected*	*Not detected*	*Not detected*
61.90	Suskie (eDNA)	1724/0.58%	46/0.02%	2725/0.91%
61.90	Suskie (sedDNA)	203/0.39%/* **0.68**	*Not detected*	219/0.43%/* **0.47**
65.60	Rekąty (eDNA)	4560/1.48%	85/0.03%	124/0.04%
65.60	Rekąty (sedDNA)	325/2.53%/* **1.71**	*Not detected*	71/0.55%/* **13.73**
69.10	Sztynorckie (eDNA)	*Not detected*	637/0.65%	*Not detected*
69.10	Sztynorckie (sedDNA)	*Not detected*	719/1.95%/* **3.02**	*Not detected*

## Discussion

### Composition of the Datasets

Both prior and after dataset harmonization, metabarcoding recorded higher numbers of taxa than microscopy, a finding mirrored across a range of organisms [78], environments [67], and environmental gradients [9]. We hypothesised that higher diversity would be observed in sedDNA due to extra reads from benthic or sediment dwelling taxa. Inspection of the top matching BLAST accessions from sediment-exclusive ASVs indicated that 6 of the 20 of the top matches – which totalled between 0% and 7.89% with a mean of 1.14% relative abundance in sediment samples – were isolated from sediments or biofilms, supporting our assumption. All 20 sediment-exclusive ASVs, made up an average of 2.39% of total relative abundance in sediment samples (0% – 9.85%).

Interestingly, one of the ASVs from sediments appearing in 16 of 22 sediment samples and consisting of a up to 12.2% relative abundance in Lake Zelwa, was identified using BLAST as belonging to *Cyanodorina* (Query Cover 100%, E-value 0, Per. Ident. 100%), a member of Microcystaceae first isolated from a eutrophic lake in Guizhou Province, China [82]. Cydrasil3 alignment placed the sequence within Microcystaceae, but did not give a conclusive alignment since Chen et al’s 2023 [82] paper was published after the most recent Cydrasil release; *Cyanodorina* is also absent from SILVA SSU r138.2. To further verify this taxon we queried the ASV sequence in NCBI Pebblescout, recovering 1,487 matches with 100% coverage and a PBSscore of 100 for the full 373 bp ASV across 112 projects, 86 of which were directly associated with aquatic environments based on Bioproject titles, and 30 of which refer to sediments in their Bioproject titles. Confirming that while recently described, this ASV is globally ubiquitous, appearing in Bioprojects from Europe, North America, South America, Africa, Oceana, and Asia, albeit under ‘unclassified cyanobacteria’, ‘uncultured bacteria’, or another placeholder, further highlighting some of the problems encountered when relying on taxonomic databases [16]. The sequence did appear in much lower levels in aquatic eDNA (9 of 22 samples, 0.006% – 0.2%), but was not identified using microscopy.

### Overall Cyanobacterial Community Composition, Potential Drivers, and Congruence Between Methods

To evaluate the first and second hypotheses regarding cross-method correspondence, we compared PCA structures using RV coefficients and environmentally constrained RDA structures using Procrustes analysis, finding partial agreement between cyanobacteria communities inferred by different methods along the trophic gradient. PCA results indicated the greatest congruence was found for within method comparisons at the genus level. With eDNA/sedDNA and cell density/biomass showing high levels of congruence based on RV coefficients. We postulate that the read based nature of metabarcoding [14], and the overlap between water-column and sediment-water interface derived communities results in principal components from each dataset capturing the same compositional directionality across datasets – which may be attributable (among other factors) to lake trophic status based on significant or near-significant correlations between TSI and the first two principal components, and consistent with the RDA results discussed below. In addition, suspended particulate matter [83] particularly in turbid waters [32], and sediment resuspension [84] can enhance eDNA transport and mixing, increasing correspondence between eDNA and sedDNA, especially for littoral samples subject to wave action and bioturbation.

The correspondence between cell density and biomass is partly expected since biomass is calculated directly from cell counts multiplied by geometric factors, and both are measures of population size using different units, having been found to be highly correlated [13]. Furthermore, dimensionality reduction in PCA of Hellinger-transformed data, performed on identical numbers of taxa may accentuate shared compositional gradients. However, it must be considered that when small taxa dominate numerically but make a relatively small contribution to biomass, or when larger taxa influence biomass disproportionally, these measures can become decoupled due to multiple order of magnitude differences between different cell volumes [85]. Ecological processes may have also contributed to the correspondence, since as lakes shift along trophic gradients the relative abundance of picocyanobacteria in both eDNA and cell-count datasets is reduced [9], decreasing picocyanobacteria influence [86, 87]. At the same time, as the relative abundance of picocyanobacteria declines, larger more colonial taxa become more prevalent, increasing the cell density and biomass of taxa better adapted to elevated nutrients [88]. Therefore, the observed correspondence between microscopy-derived datasets is likely a combination of mathematical dependence and ecological processes, and cannot be solely attributed to lake trophy.

Within our second hypothesis we expected that eDNA and cell density would have greater correspondence due to metabarcoding read numbers being more similar to cell numbers than biomass. Using High-Throughput Sequencing (HTS) derived metabarcoding data to assess relative abundance is susceptible to a number of biological biases, including problems quantifying total gene copy number variation between cells of different microorganisms [13, 93]. The average number of 16S rRNA gene copies per cell (gene copy number per genome × number of genome copies per cell, i.e. ploidy), typically varies less than geometric cell volume. For example, 16S rRNA gene copy number variation in cyanobacteria has been found to be quite low (between 1 and 5 per genome [94]. While ploidy levels vary due to environmental factors, growth phase, and nutrients [95, 96], freshwater cyanobacteria are generally polyploid [97]. In contrast, there is a range of up to 9 orders of magnitude when comparing cell volumes between types of phytoplankton [85], with cell volumes in our investigation ranging from 0.8 (*Anathece minutissima*) to 8220.0 μm³ cell⁻¹ (*Oscillatoria limosa*), i.e. a 10^4^ difference in volume. Consequently, it may be that shifts in composition along the trophic gradient were captured more broadly similarly between cell counts and eDNA than biomass, explaining the near, yet non-significant correspondence between these methods.

In our third hypothesis, we postulated that within analyses constrained by environmental variables, all methods will respond to increasing trophic status. However, when all environmental variables were entered into the RDA analysis, they were only able to conclusively explain the variance in the main community gradients of aquatic eDNA. In forward-selection RDA, TSI was identified as an important predictor for both eDNA and sedDNA. As an integrated trophic metric, TSI likely captures covariation of its component parameters, possibly explaining why it accounted for the largest proportion of community variance for eDNA and sedDNA. Kiersztyn et al. [40] also correlated TSI with multiple phytoplankton groups including cyanobacteria in the Great Masurian Lake System, attributing differences in the structure and function of phytoplankton to differences in trophic status.

The lack of significance for RDA models applied to microscopy suggests that the immediate ‘snapshot’ determined by this method may better integrate other variables related to physical structure (depth) or light conditions (Secchi depth). Also, it may be that while both cell density and biomass approached statistical significance (*p* = 0.086 and *p* = 0.096, respectively) the discrepancy between metabarcoding could be a result of inherent differences between the methods. For example, higher taxonomic resolution from metabarcoding – including in our study – may have allowed for increased statistical power in the RDA, since higher taxa counts have been suggested to increase the ability of the models to detect environmental gradients [89]. Additionally, eDNA persistence in the water column and sediments [90], which varies from days to years [91], may allow greater correspondence between the community signal captured and environmental variables [92].

Procrustes analysis appeared to show a similar correspondence between RDA ordinations constrained by environmental variables to the unconstrained RV coefficients between PCAs, likely for similar reasons, and may be explained by the discussion above.

### Relationships Between Biodiversity Determined By Each Method

We hypothesised that alpha diversity would be positively associated with the TSI, with positive correlations among methods due to diversity metrics derived from each measure being affected in a similar way along the trophic gradient, although the overall estimates were expected to differ to a degree between approaches. Contrary to our expectations, the lack of relationships found between measures of diversity that we did observe, may be a result of fundamentally different microbial ecologies of water and sediment, as well as significantly transformed cell densities when multiplied by biomass factors. SedDNA differed significantly from, and generally showed higher Shannon’s diversity than eDNA and cell density, although it was found to be lower than biomass. This may be as a result of the inclusion of benthic and accumulated taxa within sedimentary assemblages on the one hand [20, 23], while the transformed nature of biomass values on the other hand may have served to dampen the influence of extremely high picocyanobacteria cell numbers [93]. Thus, leading to a more evenly represented community when using biomass values, which for Shannon’s diversity had the highest median set of values, in addition to all lakes having a higher Pielou’s evenness index for biomass compared to any method. Differences between biomass- and cell-density-derived diversity estimates were also observed by Graham et al. [94].

Our moderate positive correlations for Shannon’s diversity and Pielou’s evenness between TSI/eDNA, and TSI/cell density may be partially explained by dominance of picocyanobacteria at lower trophic levels lowering the relative abundance of other taxa (Fig. 2a and c). Zuo et al. [30] also found that taxonomic diversity increased across sites along a eutrophication gradient in eastern China, while phylogenetic diversity declined. Additionally, Kuczyńska-Kippen et al. [29] demonstrated that in ponds, cyanobacterial diversity increased from meso- to eutrophic conditions, then declined in hypereutrophic ponds.

The absence of significant Mantel correlations for calculated Bray-Curtis dissimilarities, combined with near-zero Mantel *r* values (which would indicate either strong agreement or disagreement), indicates that beta diversity patterns are largely method-specific, in contrast to our hypothesis that common environmental structuring would lead to similar beta diversity patterns. Possibly suggesting that eDNA, sedDNA, and microscopy capture distinct aspects of cyanobacterial community variation across lakes. Envfit analysis (Fig. 6) revealed method-dependent differences in taxa whose distributions were significantly correlated with the primary axes of beta diversity, showing that small *Synechococcus* like picocyanobacteria strongly contributed to separation along Axis 1 for eDNA and sedDNA, and to a lesser extent cell density and biomass. This was in opposition to taxa typically associated with higher trophic status. The contribution of picocyanobacteria to differences in ASV community composition between lakes seems to have been driven by taxa assigned to *Cyanobium*. Similar associations have also been observed in contemporary [9] and paleolimnological studies [95]. With changes in relative abundance of *Cyanobium* over time having been attributed to variations in trophic status, as well as changes in lake circulation [96]. As with alpha diversity and the previously discussed ordinations, we interpret this as indicating that the main gradients in community structure for metabarcoding datasets are dominated by picocyanobacteria. The overall lack of correspondence between eDNA and sedDNA is likely attributable to differences in dominant taxa such as *Microcystis –* which had the second highest envfit r² value in sedDNA and 14th in eDNA – rather than differences in taxonomic richness. For example *Microcystis* is known to form blooms in the Masurian Lake district which when settled, could conceivably form a significant part of the biomass in sediments [97, 98]. In our samples, exceptionally, *Microcystis* made up 78.17% of reads in lake Sztynort sediments – a lake with a propensity toward summer cyanobacterial blooms as a result of anthropopressure [69] – with the next highest being lake Suksie with 29.22%, while 0.60% and 12.59% of aquatic eDNA reads in Sztynort and Suksie, respectively were assigned to *Microcystis*. Moreover, benthic colonies of this taxon are well known in highly eutrophic lakes [99].

For both microscopy methods envfit analysis showed that *Microcystis* had the strongest association with the primary ordination axis. Perhaps reflecting lower recorded abundance of picocyanobacteria using light microscopy [9], combined with larger *Microcystis* cell volumes, which increases both the likelihood of detection (i.e. cell counts) and, as logically follows, derived biomass estimates. Moreover, the order of envfit r² correlation coefficients for small-celled *Aphanocapsa* (2nd in cell density and 14th in biomass) further demonstrates uncoupling of community composition gradients, as a result of cell-to-biomass transformations. Other investigations have also shown poor correspondence between biomass and sequencing results [101].

While sedDNA did show consistently higher alpha diversity due to the inclusion of benthic taxa as we expected and the first principle component was positively correlated with TSI, compared to eDNA, it was relatively uninfluenced by the trophic gradient. When assessing relatedness of communities in lake surface sediments, Pearman et al. [25] found high degrees of ASV dissimilarity between hydrologically unconnected lakes. Given the direct physical connection of many of the lakes in our study, we assume the lack of a significant result for the sedDNA RDA was because community composition in sediments was determined by a combination of high regional dispersal and environmental filtering of the well-connected meta community. Which, in line with other findings [26, 34, 102], served, to some degree, to mask the relationship between the environmental conditions in the lakes and cyanobacteria in the sediments, since small-scale environmental variations have frequently been found to determine lake sediment microbial communities. This is apparent in the unconstrained ordination of Bray–Curtis dissimilarities for sedDNA (Fig. 6d), which showed a wider spatial dispersion of sites in comparison to eDNA. It must also be taken into account that while sediment samples have the potential to capture a wider frame of reference than water samples, variations in sediment accumulation rates – both between and within each lake – may theoretically have resulted in the upper layers of sediments analysed having represented anything between minutes to decades of sediment accumulation. While samples were taken from central cores or near undisturbed shores, away from potential sources of rapid accumulation, we cannot discount this as a limitation to our approach, nor discard the possibility that the relatively unclear results from sediments were due to the noise introduced into the captured biological signal by comparing dissimilar spans of time per sample.

### The Detection of Invasive Species Using Water or Sediment Samples

We assumed that the tendency of sediments to accumulate genetic material, alongside the often sporadic presence of invasive taxa in the establishment phase, would have increased the likelihood of identifying previously determined invasive species in sedDNA compared to either lakewater-based method. Previous investigations determined that the invasive taxa identified in the present study are more likely to be found in lakes of higher nutrient concentrations [41]. For *R. Raciborskii*, the lake with lowest TSI where this taxon was found, was the eutrophic lake Niegocin (TSI 55.10), with progressively higher relative percentages in lakes of higher TSI. Excluding lake Głebokie (TSI, 46.30), which only had 3 reads for *Sphaerospermopsis aphanizomenoides*, the other two lakes where it was identified were highly eutrophic. While *Cuspidothrix issatschenkoi* appeared in 12 lakes, higher abundances were observed predominantly in more eutrophic lakes, although the relationship with TSI did not appear to be strictly monotonic. This is not surprising given that it is frequently found in moderately eutrophic freshwaters [103]. Therefore, our results hint at a general relationship between lake trophy and invasive taxa occurrence, but we cannot say so conclusively.

Several sample-to-sample comparisons had between 50 and 150 times higher relative percentage contributions in sediments than water (Table 2), although others differed only 1 or 2 fold. We hypothesise that the accumulation of DNA bearing remains of these taxa, including structures likely to persist in sediments such as akinetes – a feature of all detected invasive taxa – are the most plausible explanation for relatively elevated total abundance [42]. With respect to lakes where only eDNA samples contained these taxa, this may reflect the very early stages of colonisation, before DNA bearing sediments have had time to accumulate.

## Conclusions

Our investigations indicate that there is a degree of coherence between cyanobacteria communities determined using eDNA and sedDNA along a trophic gradient, and that this correspondence was most likely due to alterations in community composition. However, it is likely that small scale variations in lake sediment habitats, which ultimately determine sediment community composition, mean that surface sediment studies (especially using easily collected samples for heterogeneous lake shores) are best suited to targeted investigations of the sediments themselves, and cannot be used as a replacement for aquatic eDNA. With respect to the correspondence between traditional light microscopy and metabarcoding, we found a borderline association between cell density and eDNA along the trophic gradient, which we attributed to the greater similarity between taxonomic quantification for these methods, than between eDNA and biomass. Our findings indicate that the abundance of invasive taxa may have increased with trophic status, and of the invasive taxa identified, lake sediments appear to have elevated relative abundances. This suggests that they may be useful for biomonitoring, but only to the extent that the presence/absence of a given organism can be determined.

## Supplementary Information

Below is the link to the electronic supplementary material.

Supplementary Material 1

Supplementary Material 2

## Data Availability

Raw sequence data have been deposited into the NCBI GenBank under BioProject number PRJNA1337660 [https://dataview.ncbi.nlm.nih.gov/object/PRJNA1337660?reviewer=gh599kf6m7nbsf5g5ukd6c3890](https://dataview.ncbi.nlm.nih.gov/object/PRJNA1337660?reviewer=gh599kf6m7nbsf5g5ukd6c3890
