# Supplementary material for: Comparing Aquatic Environmental DNA, Microscopy and Sedimentary DNA to Investigate Cyanobacterial Community Dynamics Across a Trophic Gradient
Source: Microb Ecol. 2026 Jun 30;89:168. doi: 10.1007/s00248-026-02806-2 (PMC13542045; doi:10.1007/s00248-026-02806-2)
Supplement: Supplementary file 1 [file 248_2026_2806_MOESM2_ESM.pdf]

Supplementary Information (SI) for 'Comparing Aquatic Environmental DNA, Microscopy and Sedimentary DNA to Investigate Cyanobacterial Community Dynamics Across a Trophic Gradient'

Corresponding author: Robin Crucitti-Thoo, University of Warsaw, Poland
rm.thoo@uw.edu.pl / robin.m.thoo@gmail.com

SI Section 1 - Detailed description of DNA extraction, preparation and sequencing protocol, and bioinformatic processing

DNA extraction

DNA was extracted in batches of six from approximately 250 mg of sediment of DNA water filters using the EURx Soil DNA Purification Kit (GeneMATRIX, EURx Ltd., Gdańsk, Poland) following the manufacturer's instructions. All extractions were performed under sterile conditions and the obtained DNA was stored at - 20 ℃ until further analysis. Sediment and aquatic DNA extractions were performed separately. To check the quality of the extracted DNA, DNA was placed into 1% agarose gel with a 1µl:5µl ratio with the EURx Loading Buffer TriDye (EURx), with gels stained with 0.8 µl of Midori Green Advance Stain (NIPPON Genetics EUROPE). In addition, the concentration and purity of 1µl of the DNA extract was checked using a Implen NanoPhotometer® (Instrument Type NP80, Version NPOS 4.2d build 14900) at A230, A260, A280 and A320 wavelengths).

DNA preparation and amplification

Initial test PCR reactions using the CYA primer pair (Table SI.1.) were run with two replicates and with positive and negative controls as follows:

Initial denaturation: 94℃, 4 min.
DNA denaturation: 95℃, 20s
Annealing: 62℃, 30s
Extension: 72℃, 1min.
Final Extension:72℃, 5min.
Cycle number: 30
Expected product length: approximately 400 bp

Any negative PCRs were run at least three additional times with two repetitions per PCR cycle (minimum 6 reactions total). The positive and negative controls were DNA extracted from a *Planktothrix agardhii* CCNP 1305 culture using the same DNA extraction kits and verified for the presence of all genes used in this study, and PCR Grade Sterile Water, respectively).

Each 12.5 - 13 µl PCR reaction contained 6.25 µl FastGene® Optima HotStart ReadyMix, 4.5 µl PCR-grade water, 0.625 µl forward primer, 0.625 µl reverse primer, and 0.5 - 1.0 µl DNA template. The amplifications were conducted using primers synthesised by Eurofins Genomics Europe Shared Services GmbH. PCR products were visualised in 1% agarose gel run at 80 volts for 30 minutes with gels stained with 0.8 µl of Midori Green Advance Stain (NIPPON Genetics EUROPE) and sample bands compared to a 100 bp to 2500 bp DNA Ladder (EURX Sp. z o.o.).

Table SI.1.

Target	Primers	Sequence (5'-3')	Size (bp)	Annealing Temperature	Reference
V3-V4	CYA359F	GGGGAATYTTCCGCAATGGG	400 approx.	62	Monchamp et al. 2016 [1]
	CYA784R	GACTACWGGGGTATCTAATCCC			

Next-Generation/High-Throughput Sequencing

Next-Generation Sequencing was performed by the Genomics Core Facility CeNT University of Warsaw using a NovaSeq 6000 platform on DNA extracts. Library preparation for the Illumina High-Throughput Sequencing (HTS) was performed on DNA samples subject to the following PCR conditions using the primers below:

Full NGS seq primers:

Forward

P5 Oligo - FASTA Sequence

>eurofins_adaptor_1_CYA359-F

ACACTCTTTCCCTACACGACGCTCTTCCGATCT

Primer forward - FASTA Sequence

>CYA359F

GGGGAATYTTCCGCAATGGG

Combined Forward PCR Primer Sequence

ACACTCTTTCCCTACACGACGCTCTTCCGATCTGGGGAATYTTCCGCAATGGG

Reverse

P7 Oligo - FASTA Sequence

>eurofins_adaptor_2_CYA784-R

GACTGGAGTTCAGACGTGTGCTCTTCCGATCT

Reverse primer - FASTA Sequence

>CYA784R

GACTACWGGGGTATCTAATCCC

Combined Reverse PCR Primer Sequence

GACTGGAGTTCAGACGTGTGCTCTTCCGATCTGACTACWGGGGTATCTAATCCC

Samples were subject to the following PCR conditions. In Nuclease Free H_2O with 5X PhusionTM HF Buffer 1X, 10mM dNTPs of 200 µM each, Forward-tagged Primer (CYA359F: 5'-GGGGAATYTTCCGCAATGGG-3') 10 µM to a final concentration of 0.3 µM, and Reverse primer (CYA784R: 5'-GACTACWGGGGTATCTAATCCC-3') 10 µM to a final concentration of 0.3 µM. PhusionTM High-Fidelity DNA Polymerase 2U/µL to a final concentration of 0.02 U/µL, and finally 0.2 ng/µL of template DNA for each subsample.

Twenty cycles of PCR were performed (98° 4 min, [98° 20 s, 53° 30 s, 72° 10 s x20, 72° 5 min). PCR products were subjected to electrophoresis in the 1.5% agarose gel (Invitrogen, nr 16500500) dyed with GelGreen (Biotium nr kat. #41004). 16 out of 25 ul of each sample were used. Samples were loaded with Orange G loading dye (6X) (Thermo Scientific, nr kat. J60562.AC).

The remaining 9 ul were purified with SPRI method, using KAPA Pure Beads (KAPA Biosystems nr kat. 07983271001) in the 1:0.8 ratio (sample to beads). Library amplification was performed with KAPA Universal Adapter and KAPA UDI Primer Mixes (KAPA Biosystems, nr kat. 9063781001, 9329838001) with 8 cycles of PCR.

The quality of obtained libraries were analyzed using TapeStation 2200 and High Sensitivity D1000 Reagents (Agilent, nr kat. 5067-5585). Finally, the quantity was measured by qPCR with the Kapa Library Quantification kit (Kapa Biosciences, cat. no. KK4824), according to the manufacturer's protocol.

Sequencing was performed on Illumina NovaSeq 6000 using NovaSeq 6000 SP Reagent Kit (500 cycles) (Illumina, cat. no. 20028402), with pair-end mode of 2×250 cycles and standard operating procedure and 1% Phix control library addition. Blank samples were not sequenced due to lack of products indicated in the tape station analysis.

Bioinformatic Processing

Sequencing reads were processed in QIIME2 [2]. Primers were trimmed with Cutadapt [3] with a maximum error rate of 10%. After trimming, the DADA2 pipeline was implemented [4]. The first 5 reads were removed from the 5' end of each sequence, truncated based upon the MultiQC report [5] and read quality scores, and denoised with maximum expected error per read of 2. Forward and reverse sequences were merged with a minimum overlap of 10, and chimeric sequences removed with a maximum expected error value of 2. In QIIME2, a custom naive-bayes taxonomic classifier was used for assignment, trained using the CYA primer pair in the SILVA database [6] version: 138. Taxonomic assignment was done with an 0.85 confidence threshold. Following classification, ASVs were processed to remove features with less than 100 occurrences, organelles, chloroplasts, mitochondria, and non-cyanobacterial taxa.

From a total of 48 samples, 19,165,624 demultiplexed paired-end reads were obtained. Primer trimming left 18,955,586 read pairs with a median sequence length of 231 and 229 forward and reverse reads, respectively. After implementation of the DADA2 pipeline 16,292,972 reads remained yielding 58,988 unclassified ASVs. Classification and filtering resulted in 782 ASVs identified as cyanobacteria obtained from 6,402,840 reads. Of the samples used in subsequent analysis, Lake Holny sedDNA had the lowest frequency (6,769), whereas eDNA from Rekąty had the highest (307,638). After collapsing the taxonomy, 102 genus level ASVs were classified.

SI Section 2: RV Coefficients:

Correspondence between methods

RV coefficients between PCs 1, 2, and 3 at the genus level (RV coeff.; p-value):

Bold = statistically significant; *italicized = borderline significant*

eDNA/sedDNA: 0.2904589; 0.02700271

cells/biomass: 0.4855551; 0.00005365811

eDNA/cells: 0.2009473; 0.1189902

eDNA/biomass: 0.1731616; 0.191655

sedDNA/cells: 0.1277619; 0.4783064

sedDNA/biomass: 0.1796344; 0.1908025

Family level RV:

cells/biomass: 0.2315561; 0.05474491

eDNA/sedDNA: 0.2463392; 0.05612213

sedDNA/biomass: 0.1429712; 0.4241781

eDNA/cells: 0.1519244; 0.2147408

eDNA/Biomass:0.1671643; 0.2414884

sedDNA/cells: 0.1128921; 0.4802069

Order level RV:

sedDNA/cells: 0.0567031; 0.760481
eDNA/cells: 0.1764484; 0.1118839
eDNA/Biomass: 0.0898659; 0.7449859
eDNA/sedDNA: 0.1422158; 0.3311766
sedDNA/biomass: 0.1004496; 0.6638175
cells/biomass: 0.1591445; 0.1795592

SI Section 3: RDA Graphs

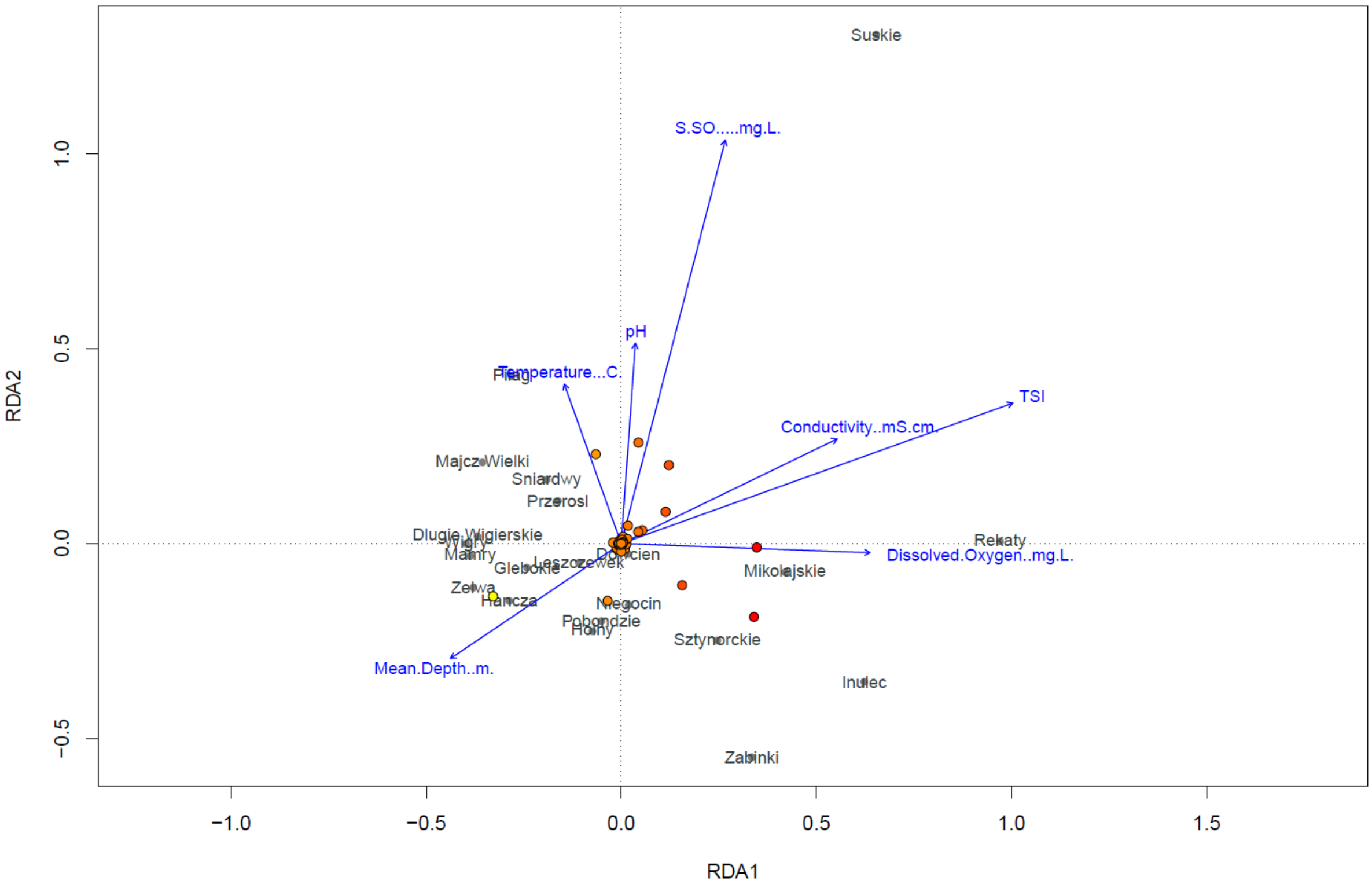

Fig. SI3.1. eDNA RDA

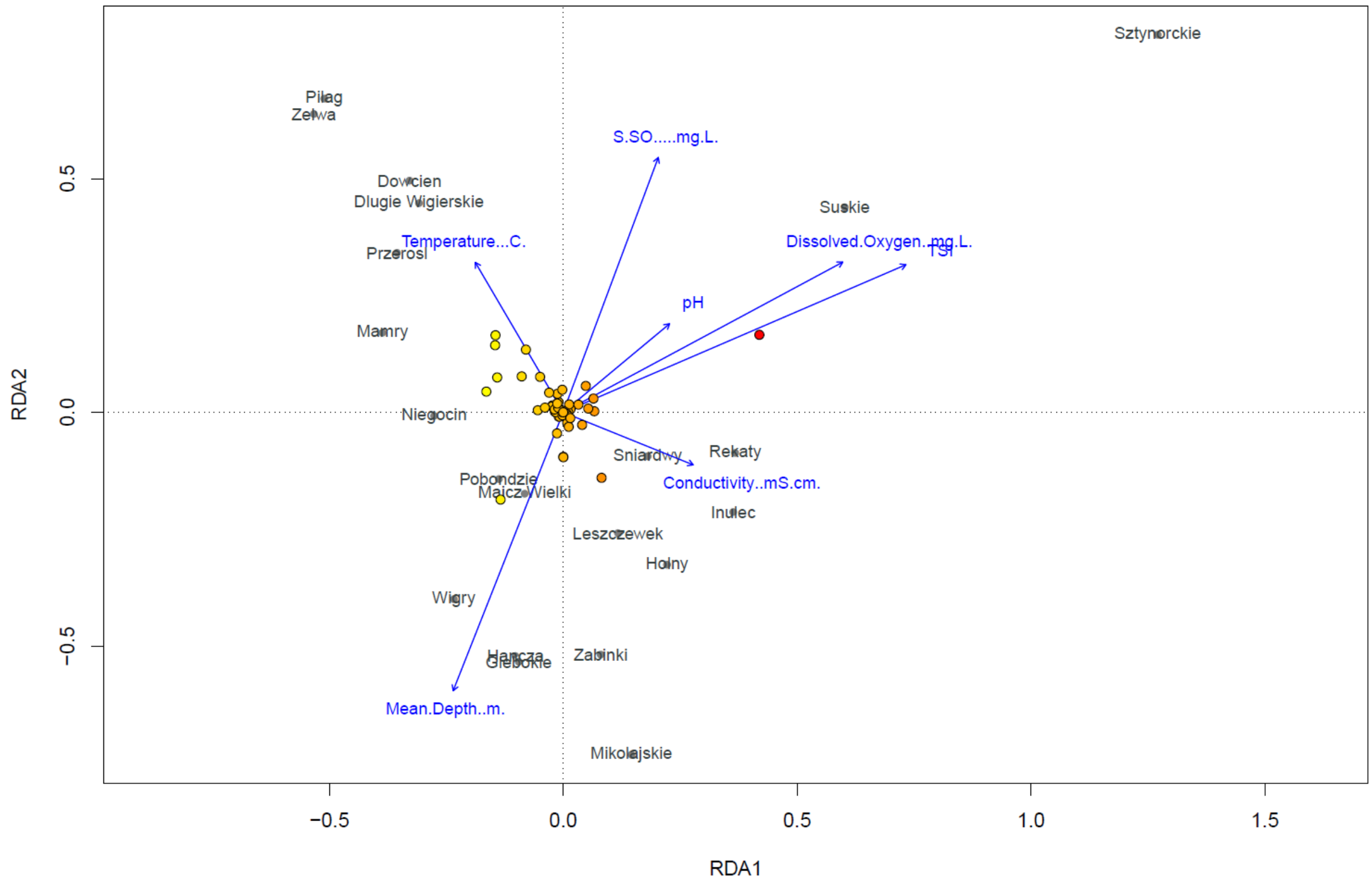

Fig. SI3.2. sedDNA RDA

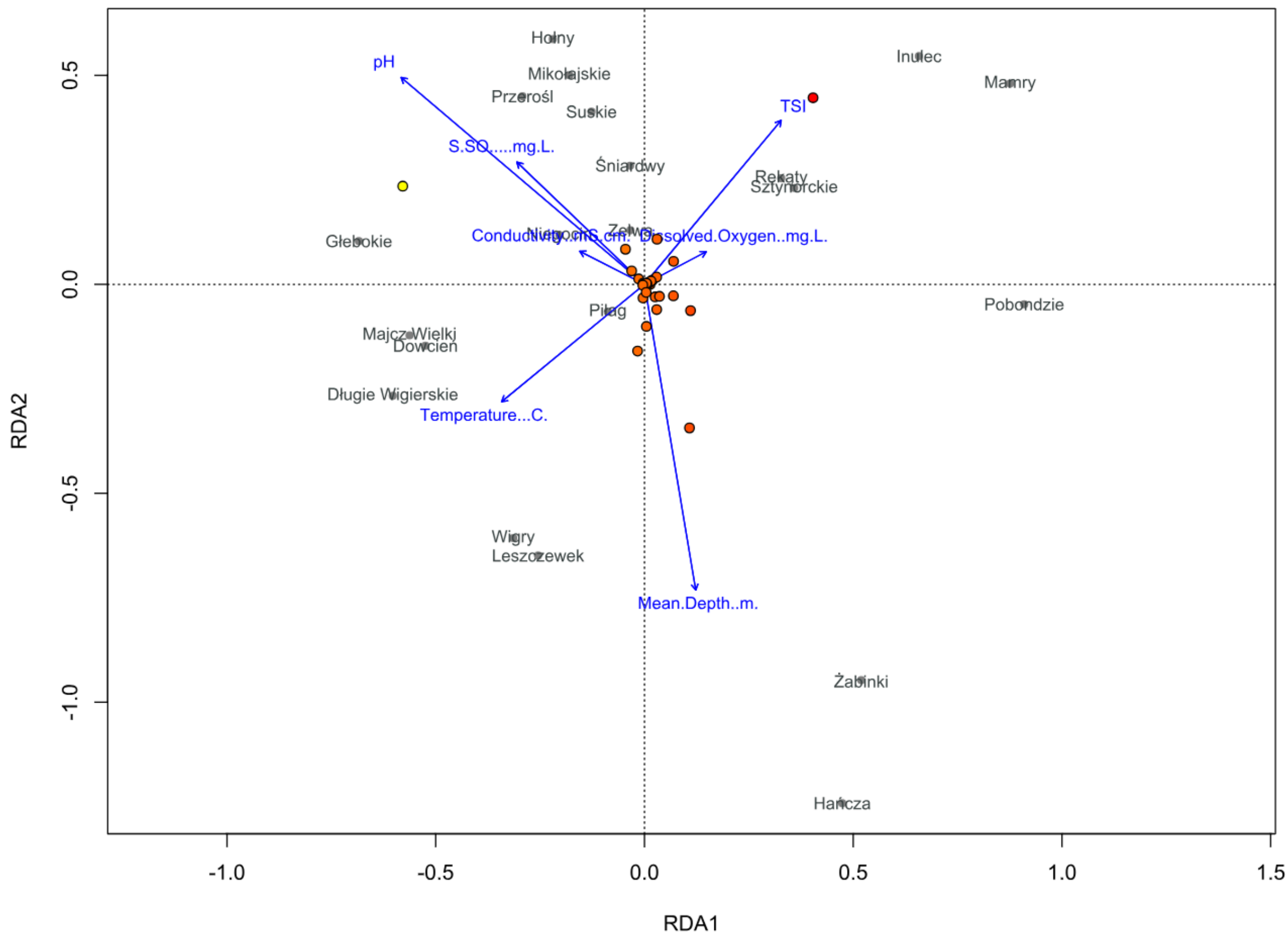

Fig. SI3.3. Cell Density (cells/L) RDA

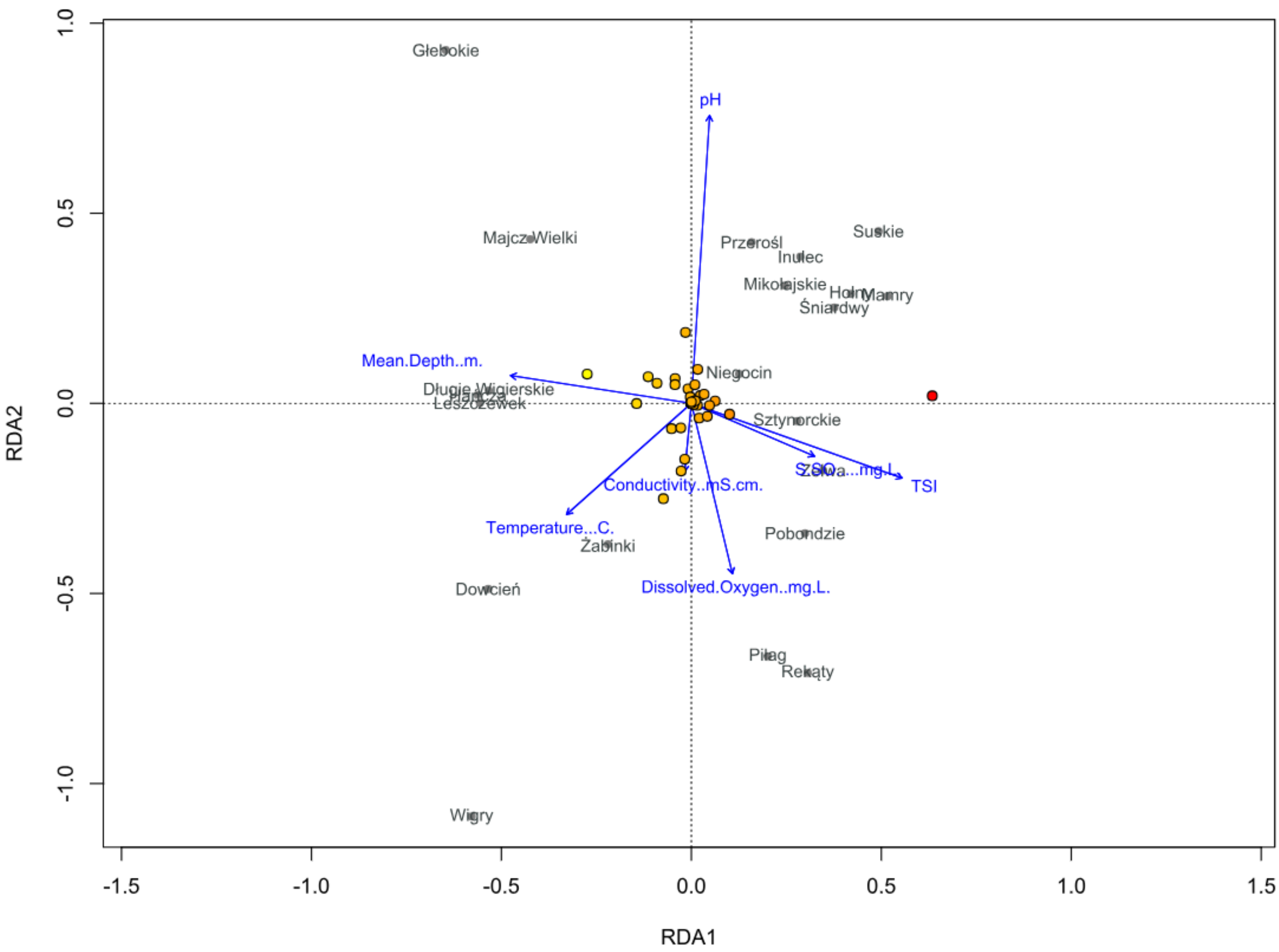

Fig. SI3.4. Biomass (mg/L) RDA

Fig. SI3: RDA graphs showing the relationship between community composition and selected environmental variables. Taxa are coloured yellow to red based on the strength of their relationship with RDA1.

SI Section 4: Procrustes Residuals

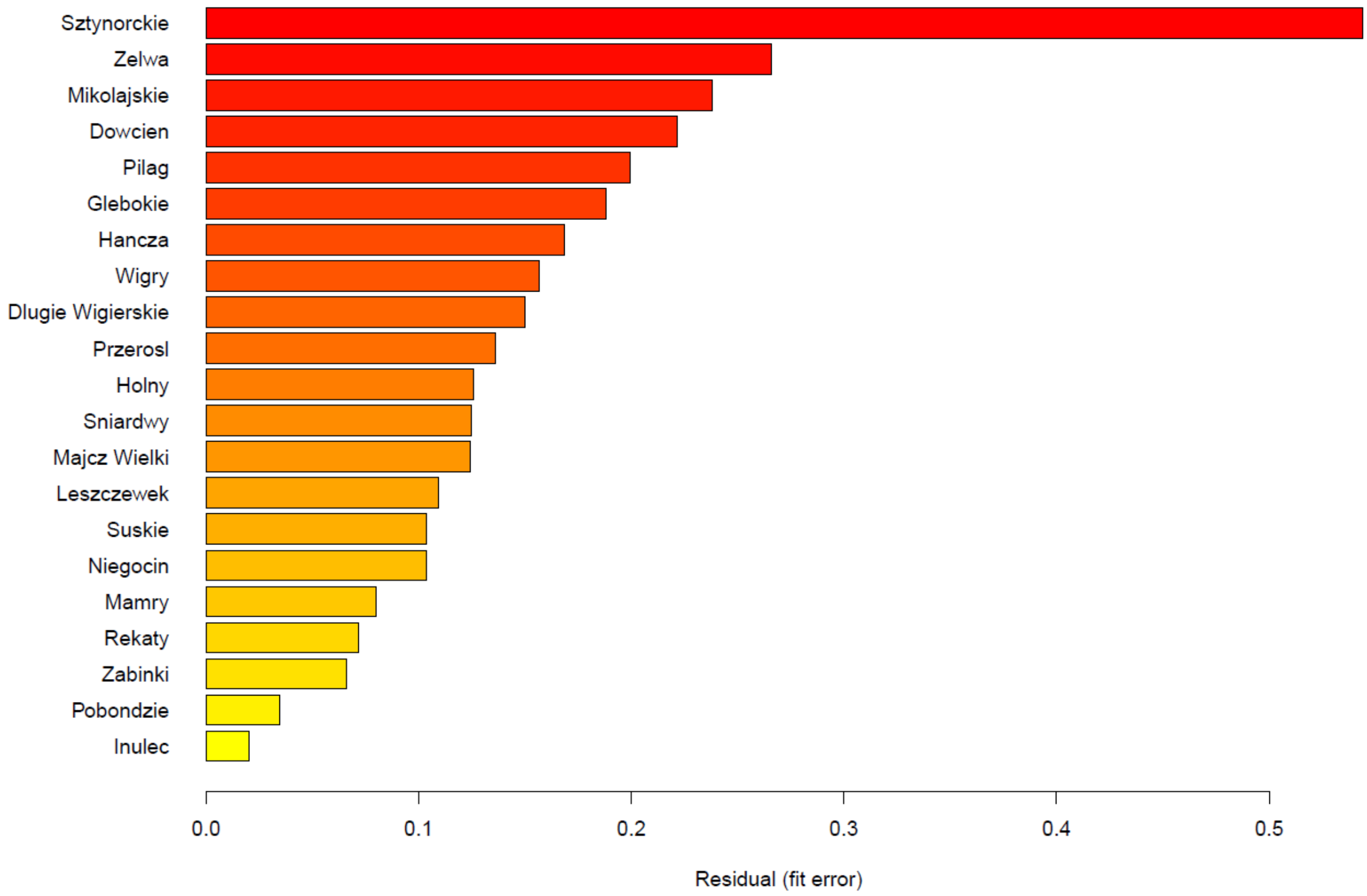

Fig. SI4.1. Procrustes residuals: eDNA vs. sedDNA

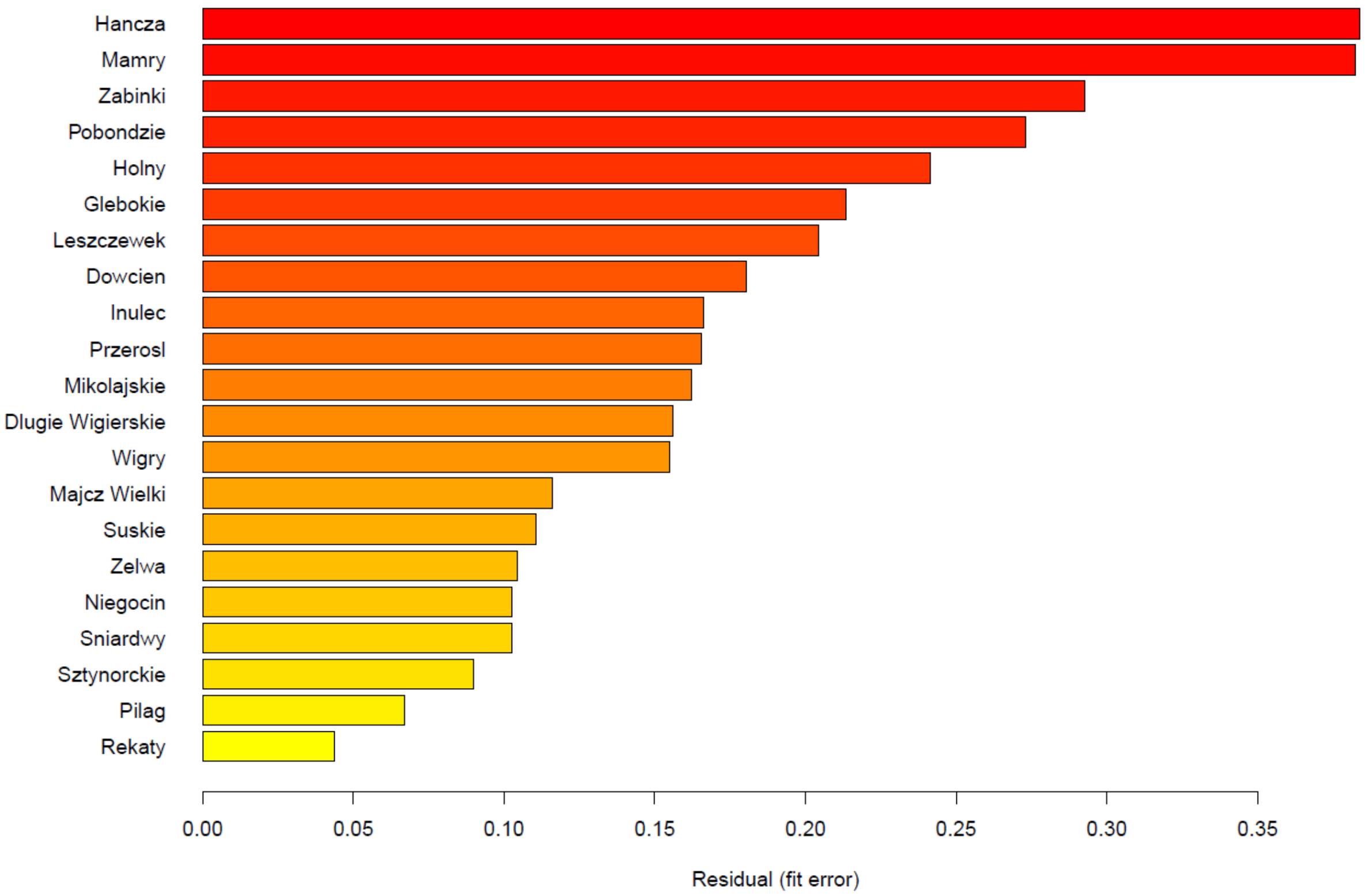

Fig. SI4.2. Procrustes residuals: eDNA vs. cell density

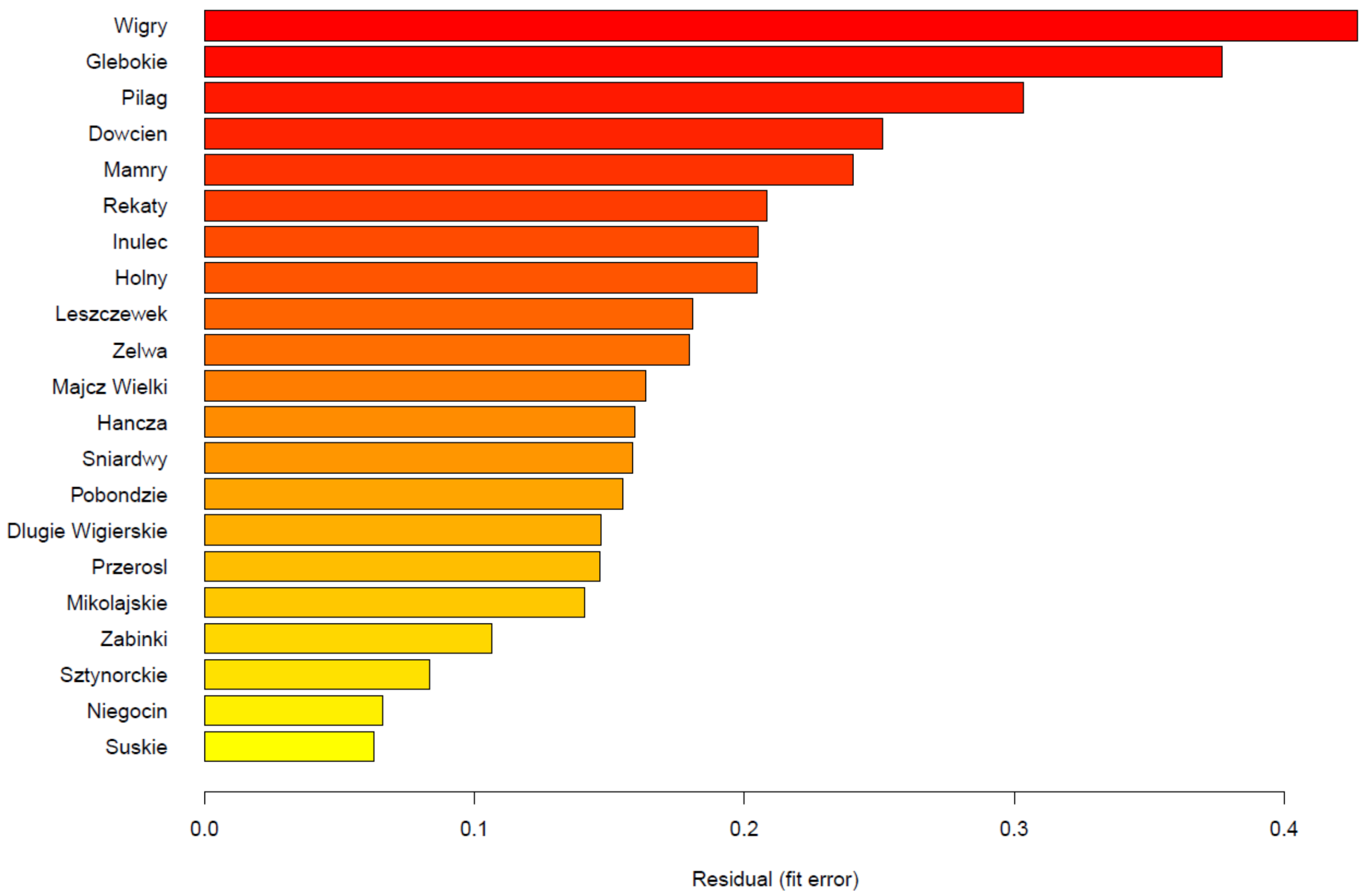

Fig. SI4.3. Procrustes residuals: eDNA vs. biomass

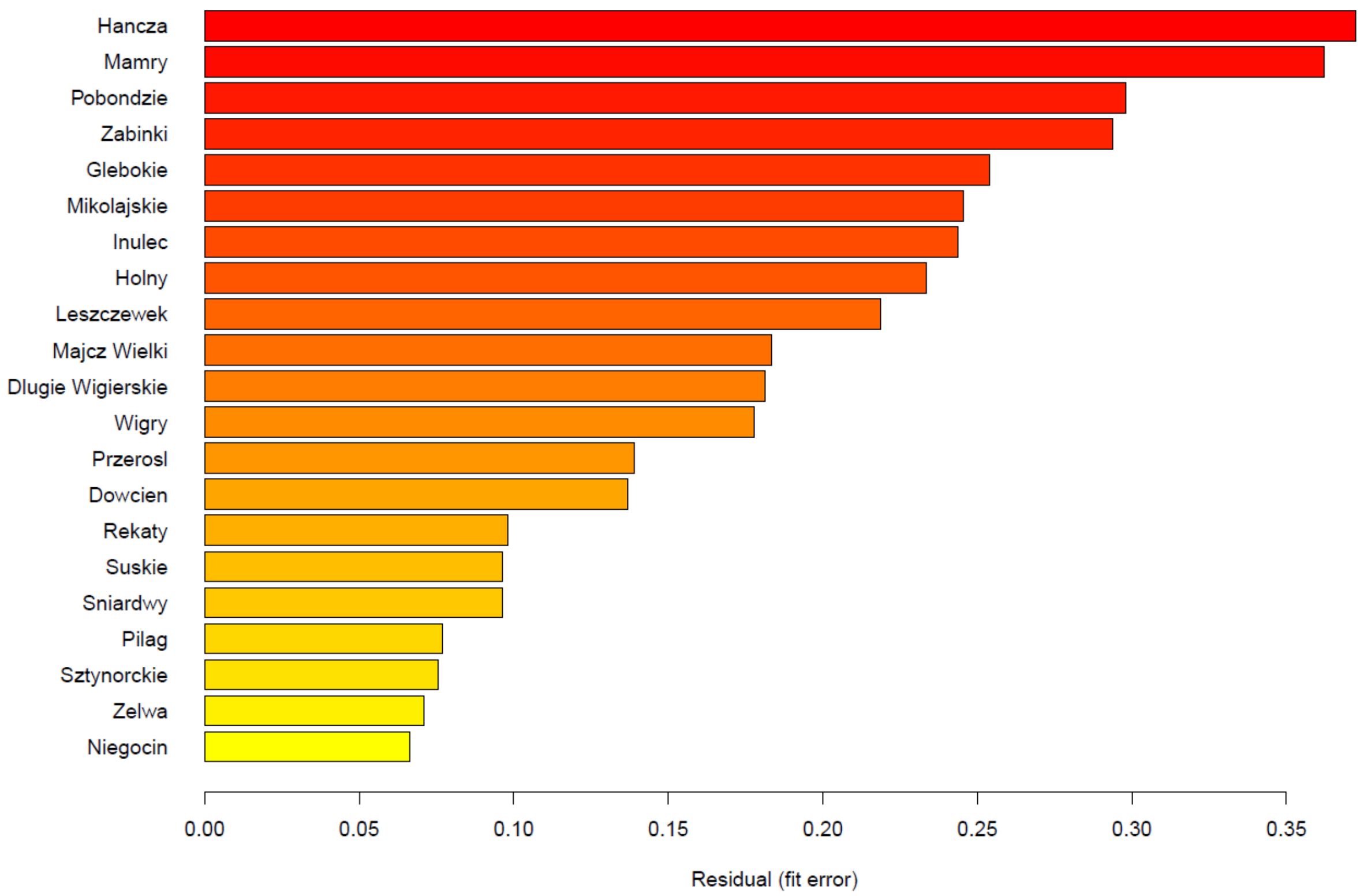

Fig. SI4.4. Procrustes residuals: sedDNA vs. cells

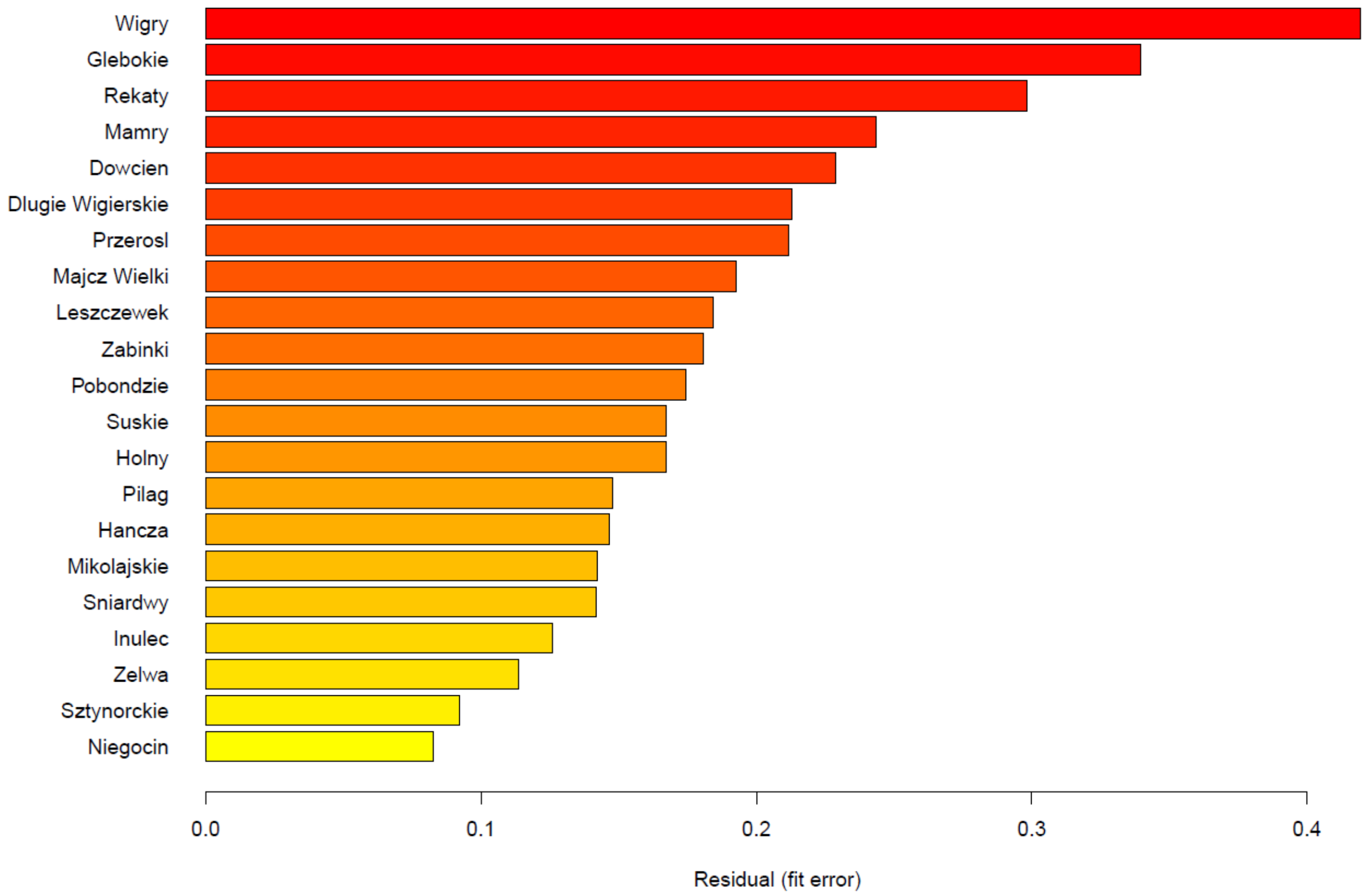

Fig. SI4.5. Procrustes residuals: sedDNA vs. biomass

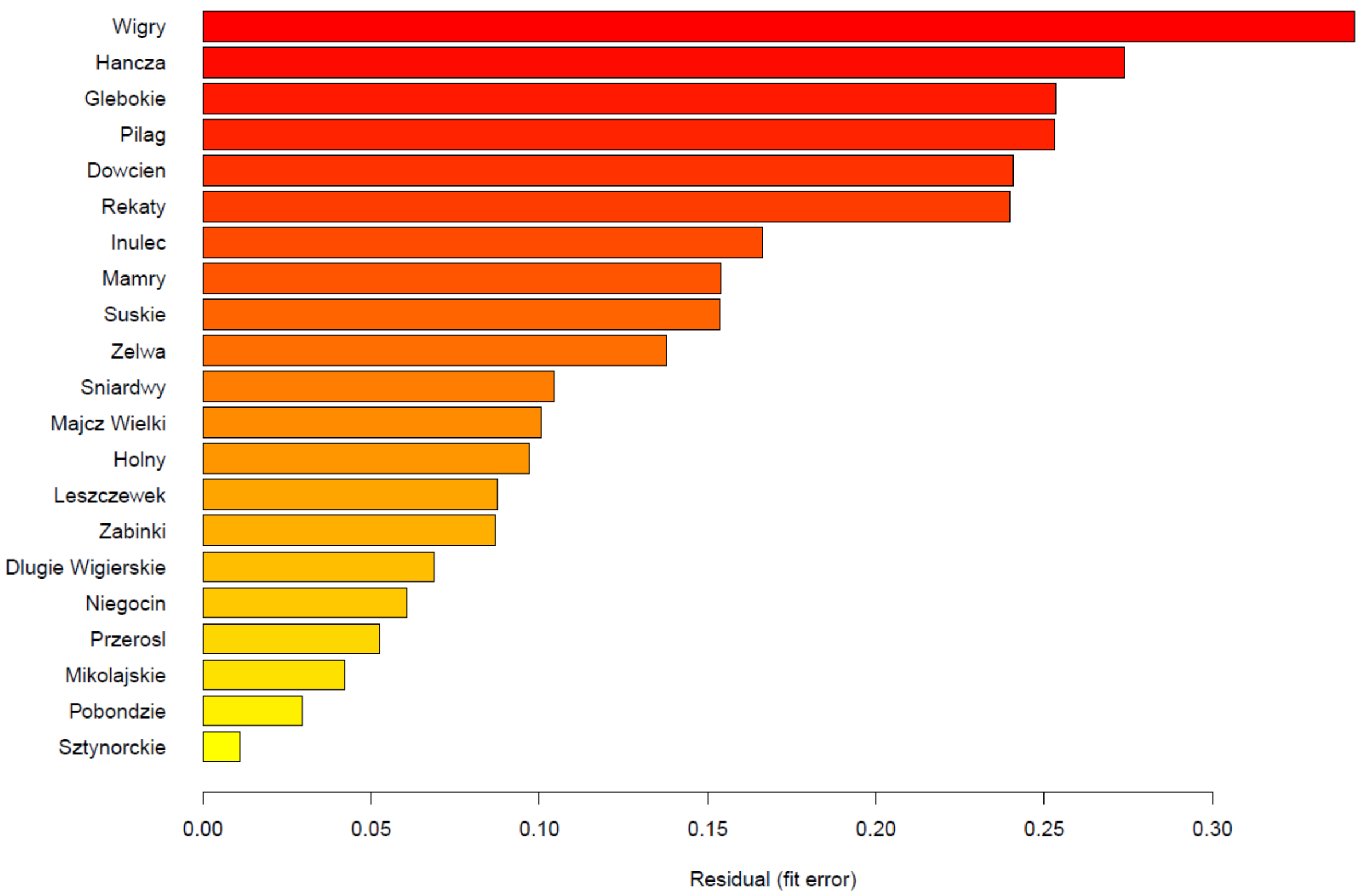

Fig. SI4.6. Procrustes residuals: cells vs. biomass

SI Section 5: Alpha diversity indices

Table SI5.1. Alpha diversity metrics

Lake	Richness_eDNA	Richness_sedDNA	Richness_cell density	Richness_biomass	Shannons_eDNA	Shannons_sedDNA	Shannons_cell density	Shannons_biomass	Pielous_eDNA	Pielous_sedDNA	Pielous_cell density	Pielous_biomass	TSI
Długie Wigierskie	14	32	9	9	0.2897551598	1.991144606	0.7176786458	2.051935945	0.1097949471	0.5745228897	0.3266296278	0.9338762938	42.9
Hańcza	13	15	7	7	0.5971959191	1.221107084	1.166469339	1.723632689	0.2328295166	0.4509174475	0.5994466595	0.8857719819	43.1
Zelwa	23	19	11	11	0.5180725588	1.840568089	1.233984184	1.786400374	0.1652283574	0.6250997566	0.5146113751	0.7449868199	43.5
Dowcień	19	31	11	11	1.033753655	2.356424309	1.337100837	1.997392427	0.3510867985	0.6862064907	0.5576143598	0.8329773402	45.4
Wigry	16	22	9	9	0.3860195357	0.9704664652	1.162245751	1.73870572	0.1392271174	0.3139608983	0.5289608366	0.791319075	45.7
Głebokie	21	18	8	8	0.4965231449	0.4242047553	0.8281231599	1.781852047	0.1630873659	0.1467647731	0.3982430587	0.8568897041	46.3
Hołny	15	21	10	10	0.4900943921	1.738003655	1.042287597	1.707998384	0.1809768489	0.5708624885	0.4526597521	0.7417742732	46.3
Majcz Wielki	23	20	9	9	0.7142568591	1.172278756	0.9908541681	2.04067986	0.2277972179	0.3913162622	0.4509571658	0.9287534287	47.1
Mamry	15	39	11	11	0.1022956105	2.130999067	1.069991394	1.723934585	0.03777463595	0.5816741391	0.4462210696	0.7189365628	47.4
Przerośl	16	33	12	12	0.7677375234	2.113732166	1.23947223	2.106244789	0.2769027794	0.6045266967	0.4988003191	0.847615257	48.7
Pobondzie	22	30	13	13	0.8272188865	1.622191757	1.53177217	2.197016053	0.2676180929	0.4769472556	0.5971939235	0.8565533845	50.7
Śniardwy	15	20	10	10	0.5522052228	1.176044235	1.40864189	1.599800422	0.2039124764	0.3925732101	0.6117653999	0.6947844953	50.8
Inulec	13	23	10	10	1.11018468	1.594589708	1.241012252	1.938979119	0.4328290836	0.5085608832	0.5389647728	0.842087932	52.2
Leszczewek	9	28	11	11	0.4646503024	1.74941483	1.64368988	2.144183943	0.2114714659	0.5250022394	0.6854719216	0.8941941575	52.4
Żabinki	13	23	11	11	1.313128912	0.9767828202	1.03901442	1.946569669	0.5119512042	0.3115243572	0.4333026685	0.8117826043	52.4
Mikołajskie	24	21	11	11	1.110220786	2.020430654	1.103438082	1.87421439	0.3493398302	0.6636281044	0.460169422	0.7816081092	54.3
Niegocin	24	41	14	14	0.7233734976	1.508622384	1.463533852	2.294050441	0.2276152439	0.4062456193	0.5545669036	0.8692688921	55.1
Piłag	20	25	9	9	0.6875404998	1.809646538	1.329021578	1.826963519	0.2295066571	0.5621983066	0.6048637868	0.8314869302	61.5
Suskie	19	28	11	11	1.898883033	2.067282878	1.178425201	1.597880023	0.6449048688	0.6203949583	0.4914414795	0.6663677273	61.9
Rekąty	24	26	14	14	1.918022084	1.714621088	1.615145458	2.019882022	0.6035209554	0.5262646664	0.6120160559	0.7653801225	65.6
Sztynorckie	15	20	12	12	0.9266316685	0.894700532	1.748514939	1.980199333	0.3421766953	0.2986583748	0.703654175	0.7968908343	69.1

SI Section 5: Alpha diversity indices

Table SI5.2. Pairwise Spearman correlations between alpha diversity metrics and trophic status index (TSI) with Benjamini–Hochberg (BH) adjusted p-values.

Metric	Comparison	Spearman's ρ	p	BH-adjusted p	n
Shannon	eDNA ~ TSI	0.609	0.003	0.034	21
Shannon	Cell density ~ TSI	0.553	0.009	0.047	21
Shannon	eDNA ~ Cell density	0.332	0.141	0.352	21
Shannon	Cell density ~ Biomass	0.344	0.127	0.352	21
Shannon	eDNA ~ Biomass	0.179	0.437	0.836	21
Shannon	Biomass ~ TSI	0.155	0.501	0.836	21
Shannon	eDNA ~ sedDNA	0.031	0.893	0.92	21
Shannon	sedDNA ~ Cell density	0.023	0.92	0.92	21
Shannon	sedDNA ~ Biomass	0.045	0.845	0.92	21
Shannon	sedDNA ~ TSI	-0.082	0.724	0.92	21
Pielou's	eDNA ~ TSI	0.585	0.005	0.053	21
Pielou's	Cell density ~ TSI	0.433	0.05	0.248	21
Pielou's	eDNA ~ Cell density	0.3	0.186	0.424	21
Pielou's	sedDNA ~ Biomass	-0.284	0.211	0.424	21
Pielou's	Biomass ~ TSI	-0.284	0.212	0.424	21
Pielou's	eDNA ~ sedDNA	0.129	0.579	0.709	21
Pielou's	eDNA ~ Biomass	-0.125	0.59	0.709	21
Pielou's	sedDNA ~ Cell density	-0.109	0.638	0.709	21
Pielou's	sedDNA ~ TSI	-0.125	0.588	0.709	21
Pielou's	Cell density ~ Biomass	-0.048	0.836	0.836	21

SI Section 6: *envfit()* taxa vector score tables

All tables are ordered from lowest p-values (top) to highest (bottom): $p < 0.05$ cells are green. Negative Dim1 coordinates are highlighted in blue.

Table SI6.1. eDNA *envfit()* taxa vector scores

**eDNA**				
	Dim1	Dim2	r2	pval
Cyanobium	-0.9989866541	-0.04500738793	0.9776190892	0.001
Limnothrix	0.637216577	-0.7706847825	0.8614764469	0.005
Planktothrix	0.6049023243	0.7962996785	0.827083132	0.001
Anabaena	0.4762026589	-0.8793355603	0.8102167722	0.004
Snowella	0.3669971419	-0.9302220691	0.7155574536	0.011
Limnothece	0.4523700956	-0.8918303071	0.6568766677	0.056
Pseudanabaena	0.9537024462	-0.3007517982	0.6216701144	0.002
Anabaenopsis	0.7213488213	0.6925719298	0.525413317	0.048
Cuspidothrix	0.9923300941	0.1236162783	0.5235605346	0.04
Kamptonema	0.7018366107	0.7123379619	0.5175086223	0.081
Geitleribactron	0.7115800721	0.7026050107	0.5023380699	0.079
Dolichospermum	0.2896987246	0.9571178867	0.4951299528	0.006
Prochlorothrix	0.2579742761	0.9661517856	0.4501559542	0.065
Microcystis	0.2875365104	-0.9577696775	0.3798173374	0.071
Nostoc	0.6862390976	0.7273760382	0.3251438534	0.09
Planktothrichoides	0.1748657694	0.9845922825	0.2874455186	0.134
Cylindrospermopsis	0.4549223299	-0.8905311189	0.2733238267	0.104
Chrysosporum	0.07021489283	0.9975318886	0.2651344805	0.093
Calothrix	0.3260106929	-0.9453660815	0.243251586	0.14
Leptolyngbya	0.9165545849	-0.3999096059	0.2426156707	0.102
Gloeocapsa	-0.8542304362	-0.519894568	0.1131735784	0.245
Phormidesmis	0.5731072271	0.819480388	0.0949308087	0.245
Geminocystis	-0.2465574555	-0.9691281758	0.0702384631	0.254
Scytonema	0.5225740541	0.8525938998	0.06767931924	0.335
Microcoleus	-0.7809965148	0.6245353824	0.06497964211	0.423
Pegethrix	-0.9928300449	-0.1195345218	0.0613741086	0.475
Aliterella	-0.4602270193	0.8878012676	0.05640574029	0.308
Synechocystis	-0.4877838315	-0.8729644516	0.04949279271	0.409
Spirulina	-0.1980524207	-0.9801914296	0.04868005351	0.349

Leptodesmis	-0.3875162359	-0.9218628786	0.04820492018	0.324
Shackletoniella	-0.8646196831	-0.5024269137	0.04581993027	0.268
Synechococcus	-0.09469658213	-0.9955061815	0.04250465179	0.56
Aphanothece	-0.5342563667	0.8453225033	0.04129635583	0.561
Cyanodorina	-0.4745352334	-0.8802365093	0.03994986206	0.484
Xenococcus	-0.2535009554	0.9673351362	0.03705956056	0.391
Chroococcidiopsis	-0.7408754566	0.671642433	0.03664403041	0.435
Cephalothrix	-0.4270586985	0.9042239037	0.03445316114	0.398
unclassified.Chroococcales	-0.4833990479	-0.8754001145	0.03113557242	0.596
Microchaete	-0.7918420815	-0.6107258943	0.02832429625	0.77
Trichotorquatus	-0.9251693275	0.3795546278	0.0265293997	0.616
Scytolyngbya	-0.5107589269	0.8597239781	0.02489701849	0.571
Chondrocystis	-0.5476848847	-0.836684688	0.02343648209	0.593
unclassified.Candidatus.Sericytochromatia	-0.6969591973	-0.7171107845	0.0234298222	0.702
Loriellopsis	-0.3044973092	0.9525131961	0.02096207343	0.682
Acaryochloris	-0.004042950817	-0.9999918272	0.01806001471	0.761
Gloeotrichia	-0.9814898767	0.1915140256	0.01509798267	0.848
unclassified.Candidatus.Obscuribacteraceae	-0.4944925927	-0.8691818428	0.01420926451	0.868
Vampirovibrio	-0.1638193398	-0.9864903567	0.01337974809	0.818
Romeriopsis	-0.5007562946	-0.8655883164	0.01026811668	0.899
Aphanizomenon	0.9926601244	0.1209374941	0.007124066503	0.928
unclassified.Candidatus.Melainabacteria	0.07578172022	-0.997124431	0.001560962184	0.959
Jaaginema	-0.8266477247	0.5627197697	0.000929083798	0.979

Table SI6.2. sedDNA *envfit()* taxa vector scores

	Dim1	Dim2	r2	pval
Cyanobium	-0.8139925112	-0.5808753668	0.9231664171	0.001
Microcystis	0.926435853	-0.3764526667	0.9120809108	0.001
Cylindrospermopsis	0.9506619429	-0.3102287388	0.8028764461	0.003
Geminocystis	-0.04287229931	0.9990805603	0.5085618513	0.019
Gloeocapsa	-0.1712355986	0.9852301101	0.4294582907	0.019
Limnothece	0.2716572534	0.9623940652	0.4191745387	0.026

Leptodesmis	-0.01292636045	0.9999164511	0.347284132	0.056
Synechococcus	-0.07384116736	0.9972700146	0.3396680516	0.061
Pseudanabaena	0.1712956789	0.9852196661	0.3347835208	0.052
Cyanodorina	-0.2724418871	0.9621722393	0.3116959082	0.064
Romeriopsis	-0.06970833663	0.9975674152	0.2852735787	0.066
unclassified.Chroococcales	-0.2941017938	0.9557741024	0.2767004722	0.072
Cephalothrix	0.1652007735	0.9862599578	0.2755139711	0.057
Leptolyngbya	0.9152300854	0.4029316205	0.2532639444	0.074
Zehria	-0.06489536092	0.9978920744	0.2375132087	0.092
Microchaete	-0.1589867935	0.9872807096	0.2284511241	0.098
Microcoleus	0.2309407756	0.9729678094	0.2108663668	0.068
Spirulina	-0.2051752106	0.9787252592	0.1979212646	0.109
Aphanothece	-0.2613329388	0.9652487219	0.190013414	0.132
Calothrix	-0.04787895462	0.9988531452	0.1875161257	0.102
Acaryochloris	-0.2210088388	0.9752718048	0.1715205393	0.185
Altericista	-0.06573119408	0.9978373666	0.166369607	0.106
Trichotorquatus	-0.1889989142	0.9819772963	0.155428462	0.198
Limnothrix	0.991522615	0.1299342291	0.1540768748	0.132
Geitleribactron	-0.2683850211	0.9633117255	0.1520745016	0.237
Scytonema	-0.1837476937	0.9829734407	0.1468028581	0.187
Chamaesiphon	-0.02847681278	0.9995944533	0.143235855	0.19
Dolichospermum	-0.09646923033	-0.9953359672	0.1423704281	0.224
Chondrocystis	-0.0765726504	0.9970640046	0.1397082932	0.205
Jaaginema	-0.3084774022	0.9512316712	0.1342877827	0.267
unclassified.Pseudanabaenaceae	-0.03263546967	0.9994673212	0.1331978122	0.18
Chrysosporum	0.263173442	-0.9647485369	0.1201330264	0.237
Cuspidothrix	0.1642049795	-0.9864262389	0.09421996872	0.348
Aphanizomenon	-0.4455119653	-0.8952759847	0.09313308018	0.329
Synechocystis	-0.2664812081	0.9638401142	0.08920989832	0.366
Anagnostidinema	0.01985408505	0.9998028882	0.08836905025	0.417
Planktothrichoides	0.1308366849	0.9914039348	0.07939065113	0.41
Anabaena	0.9224205859	-0.3861868235	0.07097460291	0.441

Merismopedia	0.1049196735	0.9944806997	0.06824489709	0.413
unclassified.Candidatus.Melainabacteria	0.5393897063	-0.8420562598	0.06233263212	0.504
Chroococcidiopsis	-0.18380747	0.9829622648	0.06138105216	0.505
Vampirovibrio	0.5314094899	-0.8471150772	0.0609338454	0.518
Candidatus.Pleurinema	-0.4308429797	0.9024269094	0.0564380259	0.596
Gloeobacter	-0.1807866888	0.9835223298	0.05624903959	0.572
unclassified.Pleurocapsales	-0.1807866888	0.9835223298	0.05624903959	0.572
unclassified.Candidatus.Sericytochromatia	-0.03878486421	-0.9992475841	0.05382383837	0.568
Anabaenopsis	0.6156459969	-0.7880228464	0.05312113127	0.586
Xenococcus	-0.210356999	0.9776246381	0.04942202265	0.611
Snowella	-0.2714432907	-0.9624544352	0.02764979009	0.811
Prochlorothrix	0.744250019	-0.6679011224	0.02748766247	0.76
Phormidesmis	-0.9955896999	-0.0938144421	0.02235728405	0.728
unclassified.Candidatus.Obscuribacteraceae	0.0294730092	-0.9995655765	0.01263316738	0.882
Nostoc	0.9960713297	0.08855453757	0.009342012998	0.924
Planktothrix	0.7788836071	-0.6271684994	0.009252079138	0.902
Chroococcopsis	0.8828616233	0.4696332123	0.009231964865	0.918
Scytolyngbya	0.8876863331	-0.460448666	0.006145735371	0.948
Pegethrix	-0.9911876296	-0.1324654024	0.00121154185	0.993
Kamptonema	-0.8499974112	-0.5267868649	0.0008039323476	0.995

Table SI6.3. Cell density (cells/L) *envfit()* taxa vector scores

	Dim1	Dim2	r2	pval
Microcystis	-0.3442729192	-0.9388696167	0.9648308511	0.001
Aphanocapsa	0.9756425097	0.2193665726	0.9551121893	0.001
Merismopedia	-0.6018199258	0.7986318156	0.9513482474	0.001
Aphanizomenon	-0.998747974	-0.05002483838	0.4822151374	0.008
Limnothrix	-0.9907058088	0.1360220588	0.4628801058	0.008
Anagnostidinema	-0.9867726719	-0.1621101292	0.3903353631	0.021
Synechococcus	-0.6683108717	0.7438821001	0.3235175094	0.028

Chrysosporum	-0.9979413638	0.06413294317	0.3168513629	0.039
Chroococcus	-0.5974817275	0.8018825259	0.3133326545	0.03
Jaaginema	-0.7496749284	0.6618062419	0.2900642979	0.054
Romeria	-0.3827954054	-0.9238331438	0.2898647089	0.14
Pseudanabaena	-0.5189174243	-0.8548243719	0.268614812	0.072
Planktolyngbya	-0.7729732796	-0.6344385778	0.268540041	0.051
Anathece	0.6806985559	-0.7325636327	0.2620715163	0.054
Spirulina	-0.5142307398	-0.8576518794	0.2329629991	0.198
Planktothrix	-0.8964804779	-0.4430832345	0.2242973816	0.089
Snowella	-0.9117345375	0.4107799085	0.1732043686	0.182
Raphidiopsis	-0.6637316245	-0.7479708087	0.1665928765	0.186
Sphaerospermopsis	-0.9301084474	-0.3672850067	0.1486367489	0.226
Umezakia	0.9981120699	0.06141901916	0.09880095173	0.263
Limnococcus	-0.08597341356	0.9962974316	0.07804964979	0.419
Leptolyngbya	-0.7028511915	0.7113369122	0.07616912119	0.478
Anabaenopsis	-0.9971647972	-0.07524870263	0.07561051749	0.537
Dolichospermum	-0.4822878122	-0.8760128231	0.07231709904	0.512
Anabaena	0.8904083144	-0.4551626453	0.06935802822	0.482
Oscillatoria	0.8904083144	-0.4551626453	0.06935802822	0.482
Cyanocatena	0.8999732738	0.4359450728	0.02519335178	0.897
Nodularia	0.8999732738	0.4359450728	0.02519335178	0.897
Woronichinia	0.9627650012	-0.2703396984	0.01214518095	0.941

Table SI6.4. Biomass (mg/L) *envfit()* taxa vector scores

	**Dim1**	**Dim2**	**r2**	**pval**
Microcystis	0.9520556143	0.3059250029	0.9310899527	0.001
Anagnostidinema	-0.4143051168	0.9101380501	0.7051714895	0.001
Merismopedia	-0.02733060317	-0.9996264493	0.6237415283	0.001
Aphanizomenon	-0.7183425377	0.6956895849	0.5703947869	0.002
Chroococcus	-0.514933244	-0.8572302807	0.4815844413	0.005
Anabaenopsis	0.3348098819	-0.9422857014	0.4598400765	0.005
Synechococcus	-0.9987345441	0.05029225031	0.4186037685	0.009
Limnothrix	-0.8948879485	0.4462908913	0.2522945026	0.089
Planktothrix	-0.2339022786	0.9722601113	0.180185825	0.162
Leptolyngbya	-0.5697051136	0.8218491854	0.1745080665	0.116
Dolichospermu	-0.7059251182	-0.7082864728	0.1413891265	0.278

m				
Pseudanabaena	0.8549694867	0.5186782979	0.1297554475	0.294
Planktolyngbya	-0.1549413049	0.9879236772	0.1244778221	0.278
Aphanocapsa	-0.9407741125	-0.3390340238	0.1187427546	0.344
Romeria	0.604942159	0.7962694169	0.1177075123	0.222
Umezakia	-0.9995635881	0.02954036842	0.1028795072	0.349
Woronichinia	-0.5286277524	-0.8488537562	0.09089996211	0.416
Raphidiopsis	0.9964000446	0.08477588741	0.08970161256	0.444
Spirulina	0.08711687274	0.996198098	0.07930274594	0.539
Nodularia	0.585178098	-0.8109047994	0.06674184236	0.672
Cyanocatena	0.585178098	-0.8109047994	0.06674184236	0.672
Jaaginema	-0.9780629104	0.2083097293	0.06653793699	0.63
Limnococcus	-0.2619942577	-0.9650694322	0.05455284243	0.656
Sphaerospermopsis	0.9142109906	-0.4052385282	0.04232696874	0.922
Anathece	-0.9946160625	0.1036286068	0.03420386487	0.727
Chrysosporum	0.9894837844	-0.1446438398	0.01357449475	0.902
Snowella	-0.9905341845	0.1372662716	0.01189714983	0.885
Anabaena	0.2943700501	0.9556915159	0.01012204294	1
Oscillatoria	0.2943700501	0.9556915159	0.01012204294	1

SI Section 7: Confirmation of Invasive Taxa

Assignments were evaluated using an integrative approach combining SILVA classification, BLAST identity, and CydraSil phylogenetic placement using the Cydrasil3 database [7] with PaPaRa: 2.5 alignment algorithm [8], EPA-ng:0.3.6 placement [9], SSU-ALIGN [10] and tree visualisation with RAxML [11]. The sequences used for invasive species can be viewed online on the iTOL website: https://itol.embl.de/tree/2128710254397241772449359

Taxon 1: For the ASV classified as "**s__Cuspidothrix_issatschenkoi**" by SILVA, 11 sequences were collapsed into a single species-level ASV. The most abundant representative sequence was queried using BLAST. The top seven hits showed 100% coverage, E-value = 0.0, and 100% identity, six of which were annotated as *Cuspidothrix issatschenkoi* and one as *Aphanizomenon* sp. NIES-3732. Cyrdasil placement is shown in Table SI.5.1 and Fig. SI.5.1

Table SI.7.1 *Cuspidothrix issatschenkoi* placement in Cyrdasil phylogenetic tree

edge_num	like_weight_ratio	distal_length
335	1	0.00005

Node 335 corresponds to: CY-NCBI-GU197654.1#g Sphaerospermopsis.s aphanizomenoides.str CHAB1306. Phylogenetic placement in CydraSil resulted in a likelihood weight ratio of 1.0 to the nearest available reference (*Sphaerospermopsis aphanizomenoides*), although *Cuspidothrix issatschenkoi* is not represented in the CydraSil reference tree. The cumulative "like_weight_ratio"

(LWR) can be used as a strong indicator of correct assignment to a given clade, with strong confidence for taxa being a LWR > 0.7.

Given the consistent 100% identity to multiple *Cuspidothrix issatschenkoi* sequences, concordant SILVA classification, and the absence of this species from the curated phylogenetic database, this ASV was assigned to *Cuspidothrix issatschenkoi*.

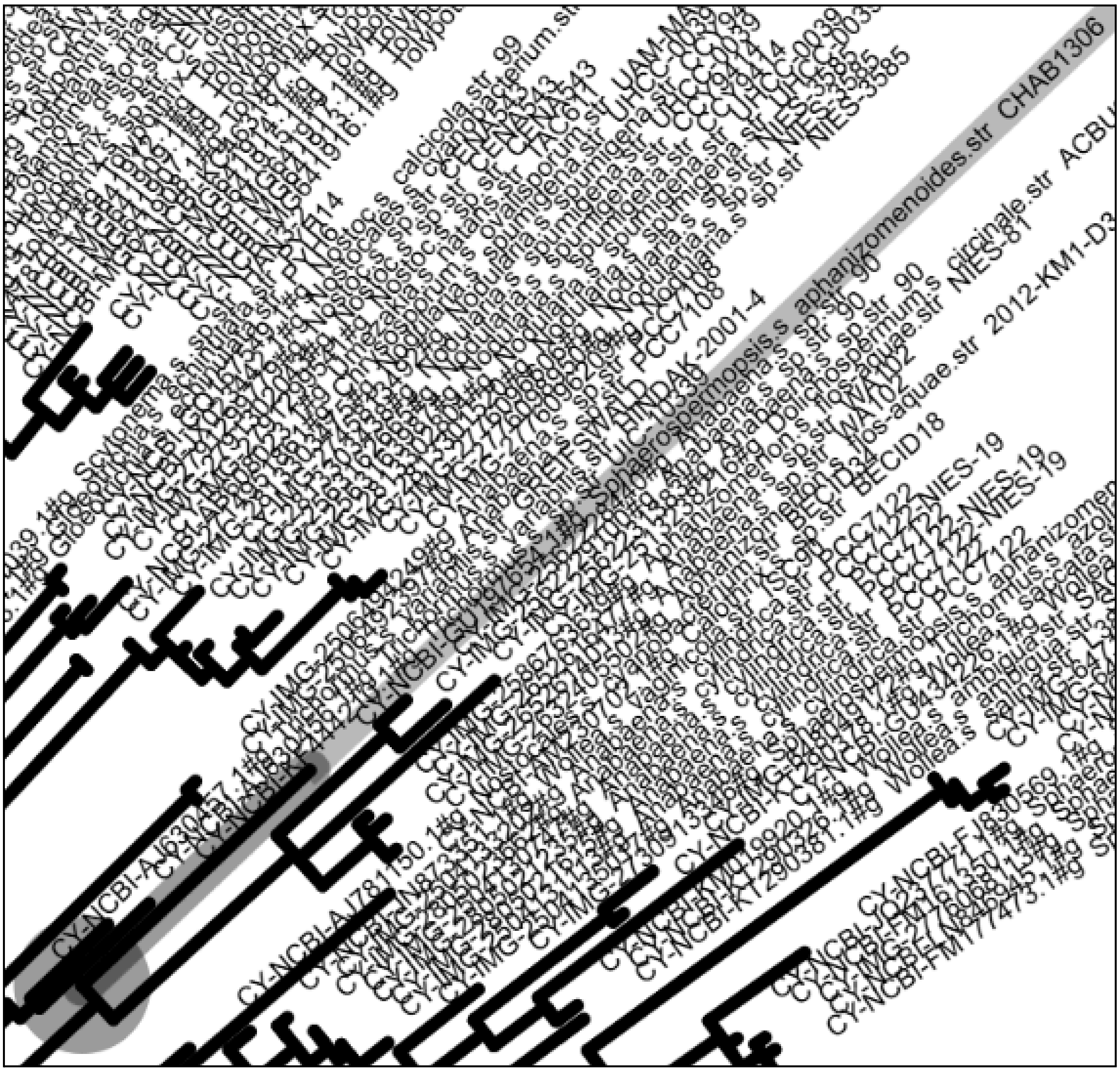

Fig SI.7.1 *Cuspidothrix issatschenkoi* placement in Cyrdasil phylogenetic tree

Taxon 2: For *Raphidiopsis raciborskii* one sequence was classified in QIIME with SILVA as "**s__Cylindrospermopsis_raciborskii**". In BLAST there were 99 hits with 100% coverage, E-value = 0.0, and 100% identity. Of which 90 were assigned to *Cylindrospermopsis raciborskii*, 2 to unspecified *Cylindrospermopsis* genera, 6 to specified *Cylindrospermopsis* genera, and three to *Raphidiopsis* genera.
Cyrdasil placements are shown in Table SI.5.2 and Fig. SI.5.2.

Table SI.7.2 *Raphidiopsis raciborskii* placement in Cyrdasil phylogenetic tree

edge_num	like_weight_ratio	distal_length
370	0.1429452488	5e-7
369	0.1429452312	5e-7

374	0.142943598	0.00005
371	0.142943563	0.00005
372	0.1429418536	0.00005
373	0.1429409927	0.00005
375	0.1423395127	0.00005

The four nodes associated with the above edges were for:

CY-IMG-647108819#g__Raphidiopsis.s__brookii.str__D9
CY-IMG-647109881#g__Raphidiopsis.s__brookii.str__D9
CY-NCBI-NZ_ACYA00000000.1#g__Cylindrospermopsis.s__raciborskii.str__CS-505
CY-NCBI-LUBZ00000000.1#g__Cylindrospermopsis.s__raciborskii.str__ITEP-A1
With a cumulative LWR of 1.

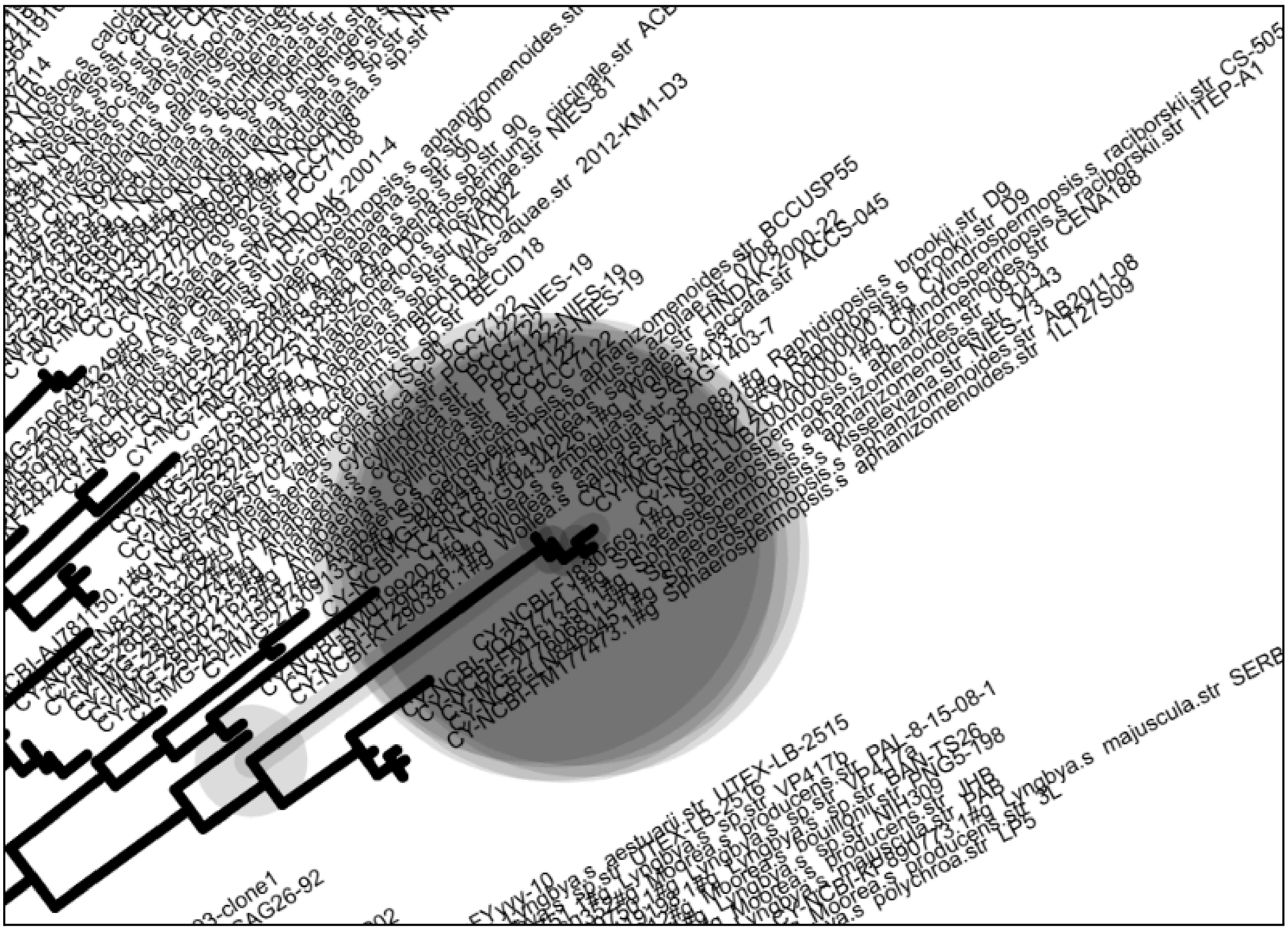

Fig SI.7.2 *Raphidiopsis raciborskii* placement in Cyrdasil phylogenetic tree

Given the strong agreement between the QIIME/SILVA classification, BLAST and Cydrasil, we are confident in assigning this ASV to *Raphidiopsis raciborskii*, noting that changes in nomenclature mean *Raphidiopsis raciborskii* is synonymous with *Cylindrospermopsis raciborskii* due to taxonomic revision.

Taxon 3: For the ASV classified in SILVA as "**s__Dolichospermum_flos-aquae**" with the genus level classification of "g__Sphaerospermopsis_BCCUSP55" which we assigned to *Sphaerospermopsis aphanizomenoides* there were 33 hits in BLAST with 100% coverage, E-value = 0.0, and 100% identity. Of which 27 were assigned to *Sphaerospermopsis aphanizomenoides*.

In Cydrasil, the sequence spread along a number of edges/nodes. 5/6 tips were labelled as *Sphaerospermopsis aphanizomenoides*, with one tip labelled *Sphaerospermopsis kisseleviana*. The cumulative LWR was 0.68. Cyrdasil placements are shown in Table SI.5.3 and Fig. SI.5.3.

Table SI.7.3 *Sphaerospermopsis aphanizomenoides* placement in Cyrdasil phylogenetic tree

edge_num	like_weight_ratio	distal_length
359	0.0968190322	5e-7
361	0.0968190322	5e-7
360	0.0968190215	5e-7
363	0.0968189849	5e-7
358	0.0968178733	0.00005
362	0.0968178135	0.00005
366	0.096817327	5e-7

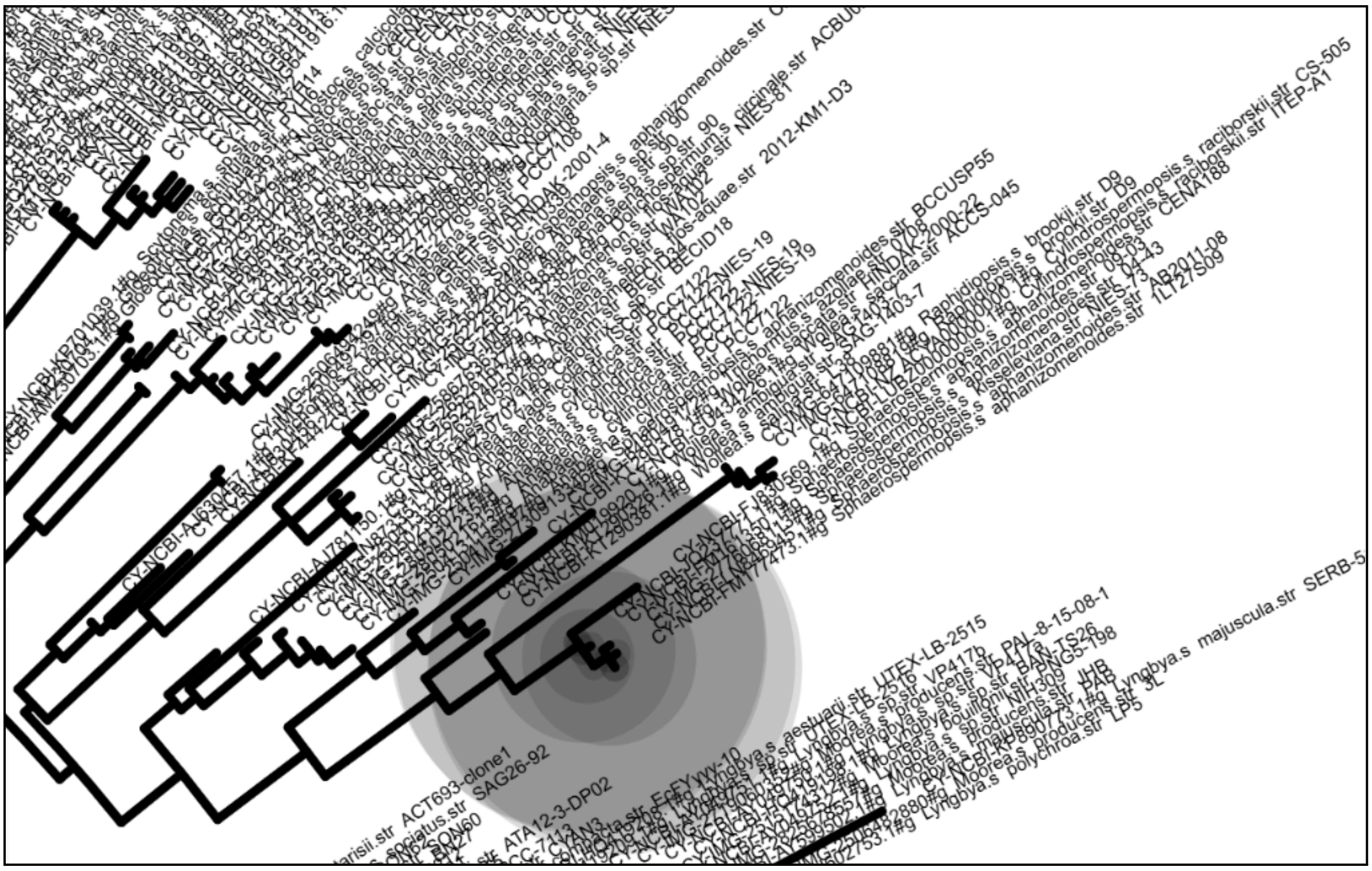

Fig SI.7.3 *Sphaerospermopsis aphanizomenoides* placement in Cyrdasil phylogenetic tree

On the basis of the above we assigned this sequence to *Sphaerospermopsis aphanizomenoides*.

References

1. Monchamp M-E, Walser J-C, Pomati F, Spaak P (2016) Sedimentary DNA Reveals Cyanobacterial Community Diversity over 200 Years in Two Perialpine Lakes. Appl Environ Microbiol 82:6472–6482. https://doi.org/10.1128/AEM.02174-16

2. Bolyen E, Rideout JR, Dillon MR, Bokulich NA, Abnet CC, Al-Ghalith GA, Alexander H, Alm EJ, Arumugam M, Asnicar F, Bai Y, Bisanz JE, Bittinger K, Brejnrod A, Brislawn CJ, Brown CT, Callahan BJ, Caraballo-Rodríguez AM, Chase J, Cope EK, Da Silva R, Diener C, Dorrestein PC, Douglas GM, Durall DM, Duvallet C, Edwardson CF, Ernst M, Estaki M, Fouquier J, Gauglitz JM, Gibbons SM, Gibson DL, Gonzalez A, Gorlick K, Guo J, Hillmann B, Holmes S, Holste H, Huttenhower C, Huttley GA, Janssen S, Jarmusch AK, Jiang L, Kaehler BD, Kang KB, Keefe CR, Keim P, Kelley ST, Knights D, Koester I, Kosciolek T, Kreps J, Langille MGI, Lee J, Ley R, Liu Y-X, Loftfield E, Lozupone C, Maher M, Marotz C, Martin BD, McDonald D, McIver LJ, Melnik AV, Metcalf JL, Morgan SC, Morton JT, Naimey AT, Navas-Molina JA, Nothias LF, Orchanian SB, Pearson T, Peoples SL, Petras D, Preuss ML, Pruesse E, Rasmussen LB, Rivers A, Robeson MS, Rosenthal P, Segata N, Shaffer M, Shiffer A, Sinha R, Song SJ, Spear JR, Swafford AD, Thompson LR, Torres PJ, Trinh P, Tripathi A, Turnbaugh PJ, Ul-Hasan S, Van Der Hooft JJJ, Vargas F, Vázquez-Baeza Y, Vogtmann E, Von Hippel M, Walters W, Wan Y, Wang M, Warren J, Weber KC, Williamson CHD, Willis AD, Xu ZZ, Zaneveld JR, Zhang Y, Zhu Q, Knight R, Caporaso JG (2019) Reproducible, interactive, scalable and extensible microbiome data science using QIIME 2. Nat Biotechnol 37:852–857. https://doi.org/10.1038/s41587-019-0209-9

3. Martin M (2011) Cutadapt removes adapter sequences from high-throughput sequencing reads. EMBnet.journal 17:10. https://doi.org/10.14806/ej.17.1.200

4. Callahan BJ, McMurdie PJ, Rosen MJ, Han AW, Johnson AJA, Holmes SP (2016) DADA2: High-resolution sample inference from Illumina amplicon data. Nat Methods 13:581–583. https://doi.org/10.1038/nmeth.3869

5. Ewels P, Magnusson M, Lundin S, Käller M (2016) MultiQC: summarize analysis results for multiple tools and samples in a single report. Bioinformatics 32:3047–3048. https://doi.org/10.1093/bioinformatics/btw354

6. Quast C, Pruesse E, Yilmaz P, Gerken J, Schweer T, Yarza P, Peplies J, Glöckner FO (2012) The SILVA ribosomal RNA gene database project: improved data processing and web-based tools. Nucleic Acids Res 41:D590–D596. https://doi.org/10.1093/nar/gks1219

7. Roush D, Giraldo-Silva A, Garcia-Pichel F (2021) Cydrasil 3, a curated 16S rRNA gene reference package and web app for cyanobacterial phylogenetic placement. Sci Data 8:230. https://doi.org/10.1038/s41597-021-01015-5

8. Berger SA, Krompass D, Stamatakis A (2011) Performance, Accuracy, and Web Server for Evolutionary Placement of Short Sequence Reads under Maximum Likelihood. Syst Biol 60:291–302. https://doi.org/10.1093/sysbio/syr010

9. Barbera P, Kozlov AM, Czech L, Morel B, Darriba D, Flouri T, Stamatakis A (2019) EPA-ng: Massively Parallel Evolutionary Placement of Genetic Sequences. Syst Biol 68:365–369. https://doi.org/10.1093/sysbio/syy054

10. E.P. Nawrocki (2009) Structural RNA Homology Search and Alignment using Covariance Models. Washington University in Saint Louis, School of Medicine

11. Stamatakis A (2014) RAxML version 8: a tool for phylogenetic analysis and post-analysis of large phylogenies. Bioinformatics 30:1312–1313. https://doi.org/10.1093/bioinformatics/btu033
